# Cardiac-derived extracellular vesicles improve mitochondrial function to protect the heart against ischemia/reperfusion injury by delivering ATP5a1

**DOI:** 10.1186/s12951-024-02618-x

**Published:** 2024-07-01

**Authors:** Xuan Liu, Qingshu Meng, Shanshan Shi, Xuedi Geng, Enhao Wang, Yinzhen Li, Fang Lin, Xiaoting Liang, Xiaoling Xi, Wei Han, Huimin Fan, Xiaohui Zhou

**Affiliations:** 1 Research Center for Translational Medicine, Shanghai East Hospital, School of Medicine, Tongji University, 150 Jimo Rd, Pudong, Shanghai, 200092 China; 2 Shanghai Heart Failure Research Center, Shanghai East Hospital, School of Medicine, Tongji University, Shanghai, 200092 China; 3 Department of Cardiothoracic Surgery, Shanghai East Hospital, School of Medicine, Tongji University, Shanghai, 200092 China; 4 Department of Heart Failure, Shanghai East Hospital, School of Medicine, Tongji University, Shanghai, 200092 China

**Keywords:** Extracellular vesicles, Myocardial ischemia-reperfusion, Mitochondria, Ferroptosis, ATP5a1

## Abstract

**Background:**

Numerous studies have confirmed the involvement of extracellular vesicles (EVs) in various physiological processes, including cellular death and tissue damage. Recently, we reported that EVs derived from ischemia-reperfusion heart exacerbate cardiac injury. However, the role of EVs from healthy heart tissue (heart-derived EVs, or cEVs) on myocardial ischemia-reperfusion (MI/R) injury remains unclear.

**Results:**

Here, we demonstrated that intramyocardial administration of cEVs significantly enhanced cardiac function and reduced cardiac damage in murine MI/R injury models. cEVs treatment effectively inhibited ferroptosis and maintained mitochondrial homeostasis in cardiomyocytes subjected to ischemia-reperfusion injury. Further results revealed that cEVs can transfer ATP5a1 into cardiomyocytes, thereby suppressing mitochondrial ROS production, alleviating mitochondrial damage, and inhibiting cardiomyocyte ferroptosis. Knockdown of ATP5a1 abolished the protective effects of cEVs. Furthermore, we found that the majority of cEVs are derived from cardiomyocytes, and ATP5a1 in cEVs primarily originates from cardiomyocytes of the healthy murine heart. Moreover, we demonstrated that adipose-derived stem cells (ADSC)-derived EVs with ATP5a1 overexpression showed much better efficacy on the therapy of MI/R injury compared to control ADSC-derived EVs.

**Conclusions:**

These findings emphasized the protective role of cEVs in cardiac injury and highlighted the therapeutic potential of targeting ATP5a1 as an important approach for managing myocardial damage induced by MI/R injury.

**Supplementary Information:**

The online version contains supplementary material available at 10.1186/s12951-024-02618-x.

## Graphical abstract

**Figure:**
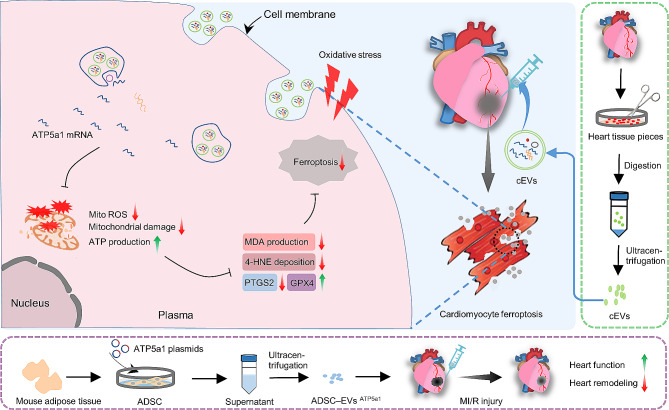
Graphical Abstract

## Background

At present, ischemic heart disease remains the leading cause of death worldwide [1]. Timely and adequate restoration of coronary blood flow is widely recognized as the most effective way to save dying myocardium and alleviate cardiac injury [2]. However, the reperfusion strategies themselves inevitably exacerbate structural and functional damages, mainly including oxidative stress, intracellular calcium overload, mitochondrial dysfunction, and the inflammatory responses, known as MI/R injury [3, 4]. Although current surgical and medical therapies can alleviate symptoms and improve their survival, patients subjected to MI/R injury progressively developed adverse ventricular remodeling and eventually chronic heart failure [5, 6]. Consequently, more efficient cardio-protective strategies are still needed.

Ferroptosis, as a newly discovered form of regulatory cell death, is triggered by the iron-dependent intracellular accumulation of lethal levels of lipid hydroperoxides [7]. Recent studies revealed that ferroptosis plays a crucial role in various cardiovascular diseases, including heart transplantation [8], atherosclerosis [9], acute myocardial infarction [10], and MI/R injury [11]. Upon ischemia-reperfusion, cardiomyocytes generate a significant amount of reactive oxygen species (ROS) and free iron. These, in turn, lead to lipid peroxidation in the cytoplasm, and ultimately cardiomyocytes ferroptosis [12]. Moreover, interventions that selectively target ferroptosis in the damaged heart have shown the potential to protect heart functions from MI/R injury [13].

Mitochondria constitute approximately 30% of the volume of cardiomyocytes and is essential for maintaining cardiac energy metabolism and normal function [14]. During MI/R injury, the disrupted homeostasis of mitochondria leads to the failure of normal lipid metabolism and iron homeostasis [15, 16]. These events inevitably result in regulatory cell death, including ferroptosis [17]. Previous studies have confirmed the significant damage of mitochondrial morphology in ferroptotic cells [18]. Further investigations have demonstrated that strategies aimed at alleviating mitochondrial damage can successfully block ferroptosis in cardiomyocytes across various settings [19, 20]. This evidence suggested the vital role of mitochondria in the ferroptosis of cardiomyocytes.

As an important mediator of intercellular communication, EVs are involved in the pathological process of diseases by transferring their cargos, such as mRNA, non-coding RNA, proteins, and other substances [21]. EVs are generally categorized into three main subtypes based on their sources: cell culture supernatant, body fluids, and tissue [22]. Compared to the former two subtypes, tissue-derived EVs offer several advantages, including tissue specificity and an accurate reflection of the tissue or organ microenvironment [23]. Cell-derived EVs have demonstrated their ability to reduce the ferroptosis of cardiomyocytes following MI/R injury by transporting effective contents [24–26]. In addition, researchers have shown that functional molecules in mitochondria, or mitochondria themselves, can be transferred by cell-derived EVs into target cells to reduce tissue ischemia injury [27, 28]. Our recent study showed that EVs derived from ischemia-reperfusion hearts harbor proinflammatory features and can exacerbate heart injury [29]. Another study demonstrated that MI/R injury causes significant adipocyte endoplasmic reticulum stress and endocrine dysfunction by releasing specifical miRNAs enriched small EVs [30]. In addition, EVs in normal tissue can participate in maintaining the physiological function of organs [20, 31–33]. Nevertheless, whether cEVs can mitigate MI/R injury and cardiomyocytes ferroptosis, and whether the mitochondrial pathway is involved in this process, need further elucidation.

The present study discovered that cEVs can attenuate myocardial damage and protect cardiac function after MI/R injury. Mechanistically, we found that cEVs alleviated mitochondrial dysfunction and protected cardiomyocytes from MI/R injury induced ferroptosis via transporting ATP5a1. Importantly, we demonstrated that ATP5a1 in cEVs mainly derived from cardiomyocytes of the healthy murine heart. Moreover, ADSC-derived EVs with overexpression of ATP5a1 exhibited a promising therapeutic effect in murine MI/R injury models. These results suggested that administration of cEVs or modulation of ATP5a1 could be considered as potential therapeutic strategies for treating MI/R injury.

## Results

### Identification and safety assessment of cEVs

**Fig. 1:**
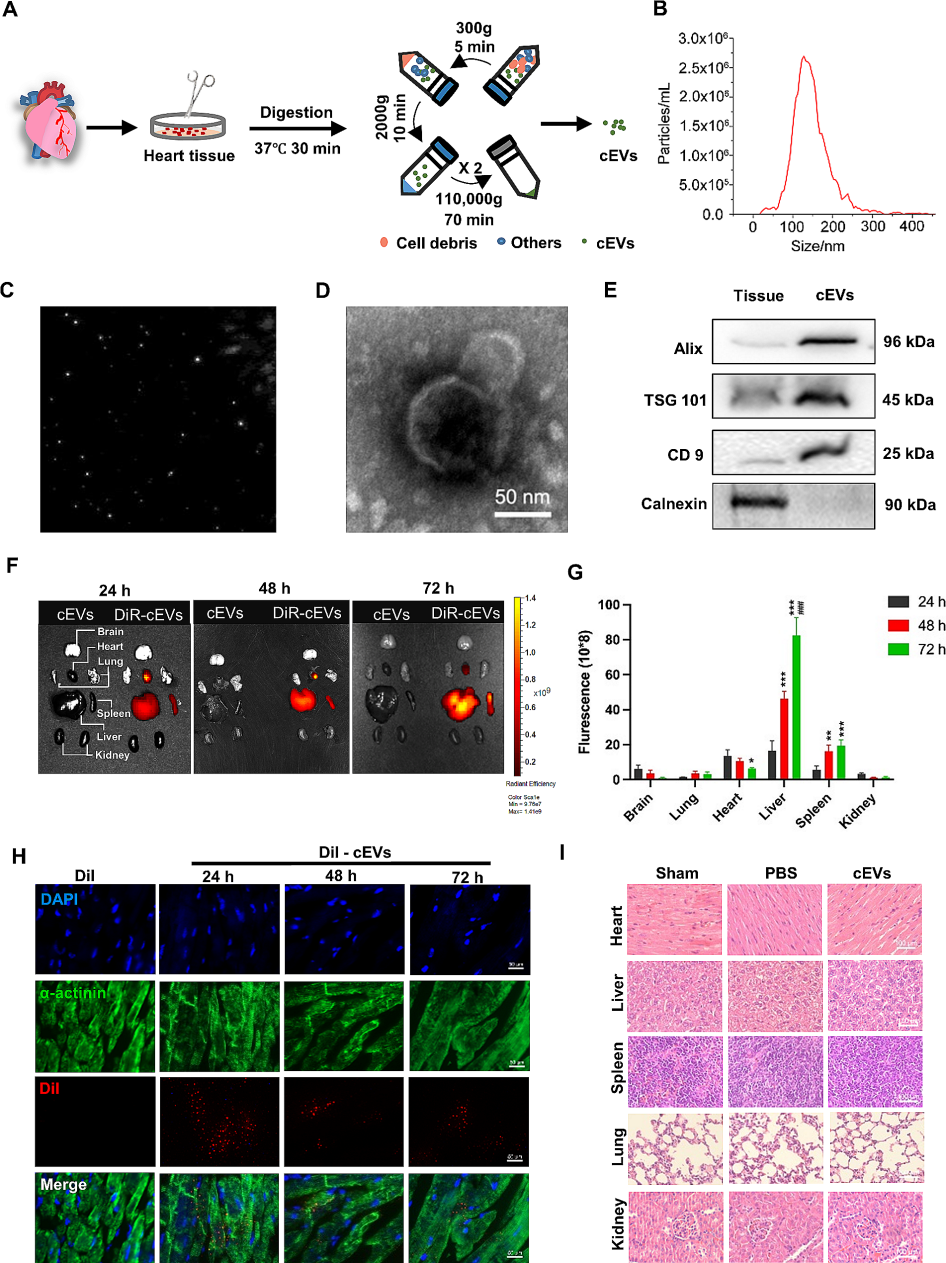
Identification and safety assessment of cEVs. **A** Schematic diagram showing cEVs isolation by a series of centrifugation steps. **B** Size distribution for cEVs detected by NTA. **C** Light scattering microscopy (LSM) images of cEVs. **D** TEM images of cEVs (scale bar = 50 nm). **E** Protein markers of cEVs were evaluated by western blot (TSG 101, Alix, CD 9, and Calnexin). **F** Representative IVIS images of different organs harvested from mice with MI/R injury at 24 h, 48 h, and 72 h after the myocardial injection of DiR-labeled cEVs. Mice that received only cEVs was regarded as negative controls (*n* = 3 in each group). **G** Measurement of the fluorescent signal in different organs of mice at 24, 48, and 72 h after the injection of DiR-labeled cEVs (*n* = 3 in each group). **H** Representative micrographs of Dil-labelled cEVs (red) in the heart at 24 h, 48 h, and 72 h after intramyocardial injection (scale bar = 50 μm). **I** H&E staining of major organs (Heart, Liver, Spleen, Lungs, and Kidneys) in mice at day 7 after myocardial injection with PBS or cEVs (scale bar = 100 μm, *n* = 5 in each group). (Data were expressed as Mean ± SD. (Statistically significant: *p < 0.05, **p < 0.01, and ***p < 0.001 for the fluorescence intensity in 48 h and 72 h vs. 24 h; ^###^*P* < 0.001 for the fluorescence intensity in 72 h vs. 48 h)

cEVs were isolated by ultracentrifugation, as depicted in Fig. 1A. NTA analysis showed that the average diameter of cEVs was 156 nm, which fell within the size range for EVs (Fig. 1B, C). In addition, Transmission Electron Microscope (TEM) micrographs revealed the typical cup shape of cEVs (Fig. 1D). Western blot analysis further confirmed the positive protein markers for cEVs, including TSG 101, Alix, and CD 9, and the negative expression of Calnexin (Fig. 1E).

Next, we labeled cEVs with DiR in vitro and assessed their biodistribution using IVIS imaging system after intramyocardial injection. As shown in Fig. 1F and G, 24 h after injection, cEVs primarily accumulated in the heart, liver, and spleen, with a small distribution in the brain, lungs, and kidneys. From 48 h to 72 h, the signal of cEVs decreased slightly in the heart while increased in the liver and spleen. No obvious signal was detected in the cEVs alone group at the same time points. Next, to better clarify the retention of cEVs in the heart and confirm their endocytosis by cardiomyocytes in vivo, cEVs were labeled with Dil in vitro and then intramyocardially injected into the heart. Subsequently, the fluorescent signal of Dil-labeled cEVs was detected in heart tissue sections. Figure 1H showed that cEVs co-localized with cardiomyocytes at 24 h, 48 h, and even persisted at 72 h after intramyocardial injection, indicating a well retention of cEVs in the heart and the uptake of cEVs by cardiomyocytes in vivo. Moreover, hematoxylin and eosin (H&E) staining showed no obvious pathological changes in murine heart, liver, spleen, lungs, and kidneys after cEVs intramyocardial injection (Fig. 1I).

### Administration of cEVs protected heart function and ameliorated adverse cardiac remodeling in mice post MI/R

**Fig. 2:**
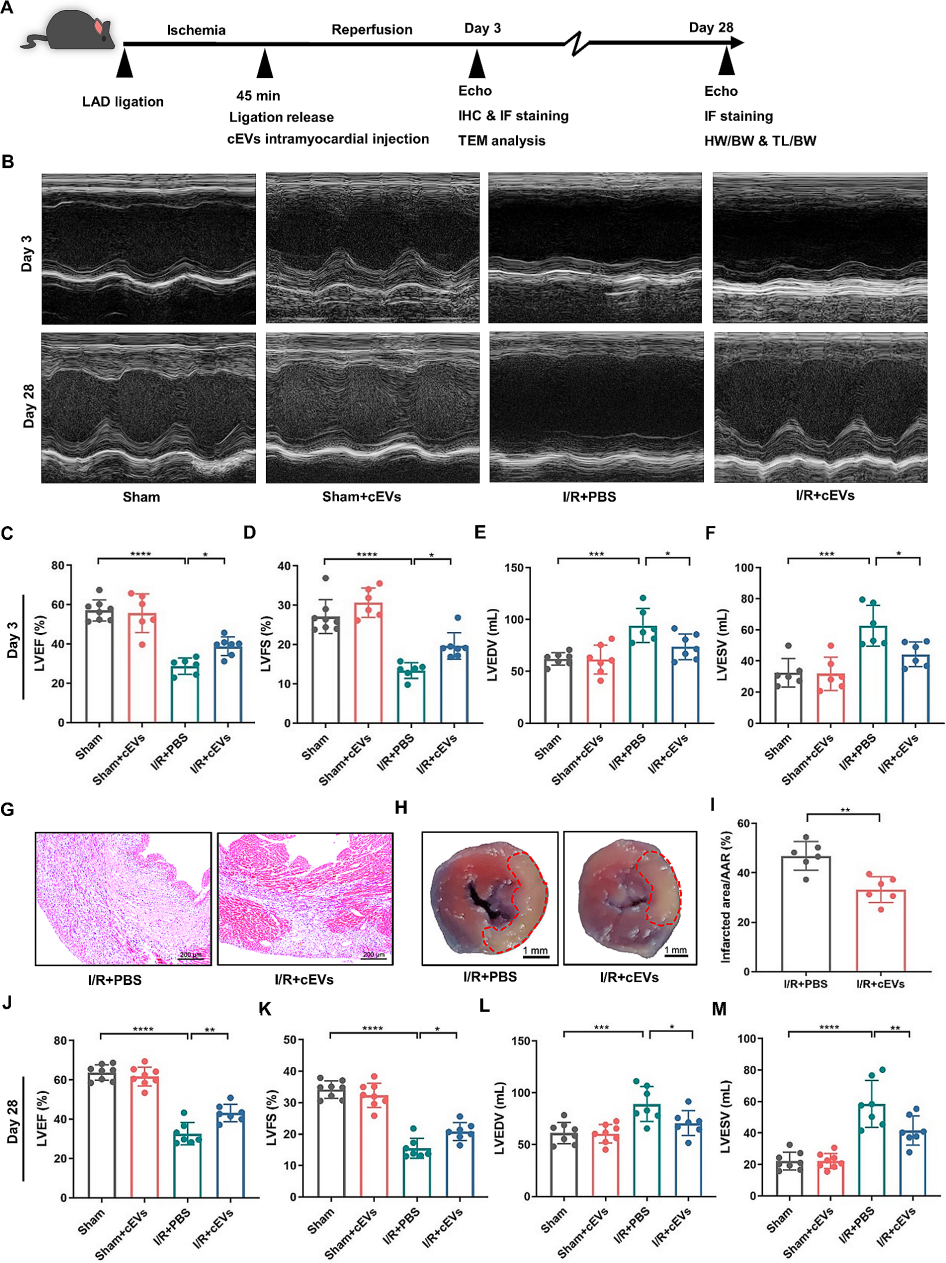
Administration of cEVs protected heart function and ameliorated adverse cardiac remodeling in mice post MI/R injury. **A** Experimental flow chart for in vivo study. **B** Representative images of cardiac function at day 3 and day 28 post MI/R determined by M-mode echocardiography (*n* = 6–8 in each group). **C-F** Statistical results of echocardiogram illustrated (**C**) LVEF, (**D**) LVFS, (**E**) LVEDV, and (**F**) LVESV in mice receiving different treatment at day 3 post MI/R (*n* = 6–8 in each group). **G** Representative H&E staining of mice heart at day 3 after MI/R injury (scale bar = 200 μm, *n* = 6 in each group). **H**, **I** Representative TTC staining and corresponding analysis showing infarcted area/AAR ratio of mice heart at day 3 after MI/R injury (scale bar = 1 mm, *n* = 6 in each group). **J-M** Analysis of LVEF (**J**), LVFS (**K**), LVEDV (**L**), and LVESV (**M**) in mice at day 28 post MI/R (*n* = 6–8 in each group). (Data were expressed as Mean ± SD. Statistically significant: *p < 0.05, **p < 0.01, ***p < 0.001, ****p < 0.0001)

Our recent study demonstrated that EVs derived from ischemic-reperfusion injured heart tissue boosted local inflammation and aggravated cardiac MI/R injury [29]. Thus, we investigated whether cEVs could alleviate the cardiac injury post MI/R (Fig. 2A). As illustrated in Fig. 2B-F, both left ventricular ejection fraction (LVEF) and left ventricular fraction shortening (LVFS) significantly increased, while left ventricular end-diastolic volume (LVEDV) and left ventricular end-systolic volume (LVESV) notably decreased in cEVs treated mice compared to the PBS control at day 3. In addition, H&E staining indicated less inflammatory infiltration in the cEVs-treated heart than in the PBS-treated heart (Fig. 2G). Triphenyl tetrazolium chloride (TTC) staining showed that the infarct area/AAR ratio was significantly lower in mice treated with cEVs than those receiving PBS treatment (Fig. 2H, I). Cardiac remodeling at 4 weeks post MI/R injury was further assessed through M-mode echocardiograph and histological examination. While the LVEF and LVFS decreased in PBS treated animals subjected to MI/R injury compared to the Sham group, cEVs treatment mitigated cardiac function damage compared to PBS-treated mice (Fig. 2J, K). Administration of cEVs also led to a decrease in LVEDV, LVESV (Fig. 2K, L), and cardiomyocytes cross-sectional area (Additional file 1: Fig. S1A, B) compared to mice treated with PBS 28 days after operation, respectively. Similarly, cardiac fibrosis at day 28 following MI/R injury was significantly lower in mice treated with cEVs compared to those treated with PBS (Additional file 1: Fig. S1C, D). Moreover, the heart weight/body weight (HW/BW) and heart weight/tibia length (HW/TL) in cEVs-treated mice were lower than those in PBS-treated mice (Additional file 1: Fig. S1E, F). Collectively, these data revealed that intramyocardial injection of cEVs could improve cardiac function and ameliorate cardiac remodeling after MI/R injury.

### cEVs alleviated ferroptosis and mitochondrial dysfunction in cardiomyocytes under oxidative stress in vivo

Then, we evaluated whether administration of cEVs affected the cardiomyocytes apoptosis post MI/R injury. Terminal deoxynucleotidyl transferase dUTP nick end labeling (TUNEL) staining results showed a significant increase in cardiomyocytes apoptosis at day 3 in the hearts treated with PBS compared to sham surgery. However, the ratio of TUNEL-positive cells in cEVs-treated mice showed no difference compared to PBS-treated mice (Additional file 1: Fig. S2A, B), suggesting that cEVs may exert cardiac-protective roles by mediating others forms of cell death.

**Fig. 3:**
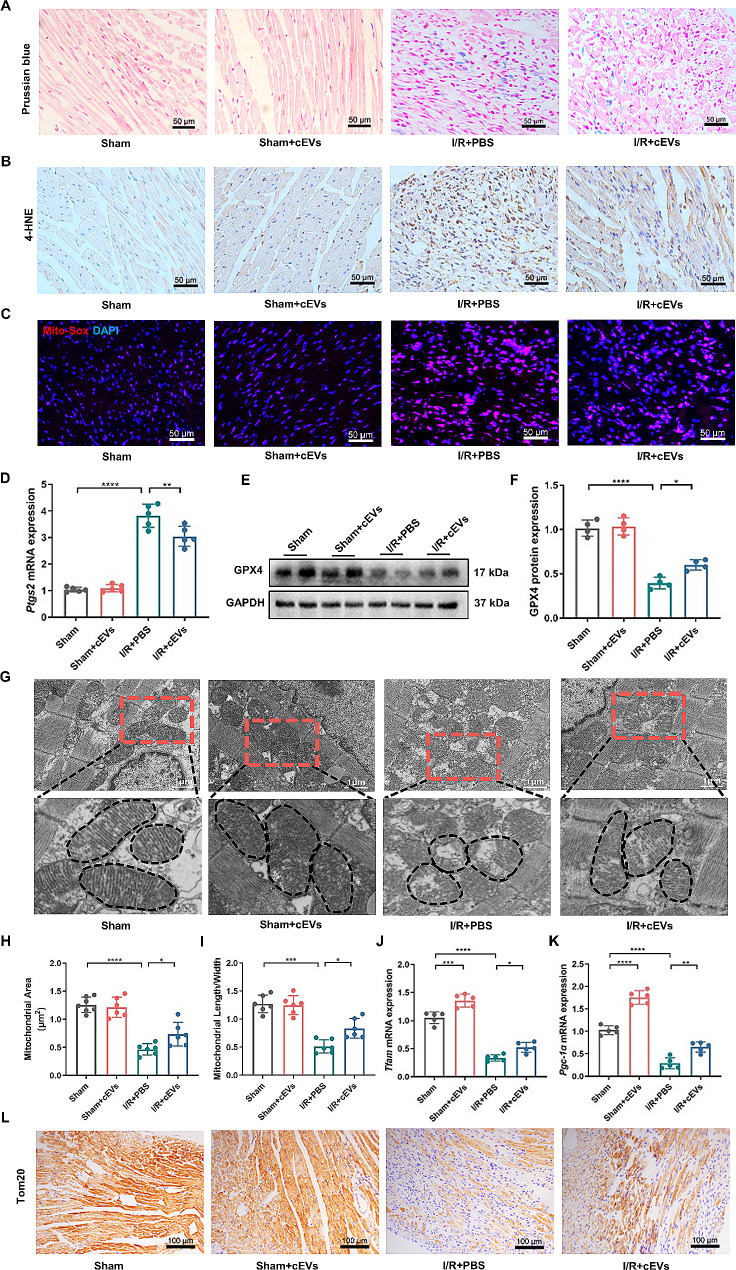
cEVs alleviated ferroptosis and mitochondrial dysfunction in cardiomyocytes under oxidative stress in vivo. **A** Prussian blue staining showing decreased iron accumulation in mice heart treated with cEVs compared to treated with PBS at day 3 after MI/R (scale bar = 50 μm, *n* = 6). **B** Evaluation of 4-HNE accumulation in MI/R mice heart of different groups detected by IHC staining (scale bar = 50 μm, *n* = 6). **C** MitoSOX staining indicating mitochondria superoxide production in mice heart (*n* = 4). **D** Relative mRNA expression of Ptgs2 in mice heart of different groups detected by RT-qPCR (*n* = 5). **E, F** Relative protein expression of GPX4 in mice heart of different groups detected by western blot and corresponding quantification analysis (*n* = 4). **G** Representative TEM images showing the mitochondrial morphology in MI/R mice heart (scale bar = 1 μm, *n* = 5). **H, I** Mitochondrial area (**H**) and mitochondrial length/width ratio (**I**) detected in TEM images. **J, K** Relative mRNA expression of TFAM (**J**) and PGC-1α (**K**) in mice heart of different groups detected by RT-qPCR (*n* = 5). **L** Representative micrographs of Tom20 IHC staining in mice heart at day 3 after operation (scale bar = 100 μm, *n* = 6). (Data were expressed as Mean ± SD. Statistically significant: *p < 0.05, **p < 0.01, ***p < 0.001, ****p < 0.0001)

Previous study revealed that ferroptosis plays an important role in the pathogenic process of MI/R injury. We, therefore, investigated the influence of cEVs on cardiomyocytes ferroptosis in murine MI/R injury models. As shown by Fig. 3A, MI/R operation led to a significant increase in iron accumulation in the heart, as evaluated by Prussian blue staining, which was remarkably alleviated by cEVs treatment. Additionally, we conducted 4-hydroxynonenal (4-HNE) staining to detect lipid peroxidation and MitoSOX staining to assess mitochondrial superoxide production in the cardiac tissue, respectively. Mice treated with cEVs exhibited reduced 4-HNE and MitoSOX signal compared to those observed in PBS-treated mice (Fig. 3B, C).

Moreover, compared to PBS treatment, cEVs administration significantly inhibited prostaglandin-endoperoxide synthase 2 (PTGS2) mRNA expression and restored glutathione peroxidase 4 (GPX4) protein expression in the heart post MI/R injury, both of which are molecular markers of ferroptosis (Fig. 3D-F). Taken together, these results suggested that cEVs treatment alleviated cardiomyocytes ferroptosis and improved cardiac function in murine MI/R injury models.

Mitochondrial dysfunction and metabolic alteration are reported to be responsible for cell ferroptosis [34]. We hypothesized that cEVs may mitigate ferroptosis in cardiomyocytes by preventing mitochondrial damage in the context of MI/R injury. TEM analysis showed obvious damage to mitochondria structure in cardiomyocytes after MI/R injury, as indicated by dissolved mitochondrial cristae and a ruptured outer membrane, which were alleviated by cEVs administration (Fig. 3G-I). Additionally, MI/R injury dramatically decreased the mRNA expression of peroxisome proliferator–activated receptor γ coactivator-1 (PGC-1α) and mitochondrial transcription factor A (TFAM), both of which are mitochondrial function-related molecular markers. As expected, cEVs treatment reversed the reductions in PGC-1α and TFAM expression in the heart subjected to MI/R injury (Fig. 3J, K). Immunohistochemistry (IHC) staining showed that Tom20 protein expression in the hearts of mice treated with cEVs was significantly higher than that in the PBS-treated group (Fig. 3L). These results demonstrated that administration of cEVs attenuated mitochondrial damage in cardiomyocytes post MI/R injury in vivo.

### cEVs attenuated oxidative stress-induced cell ferroptosis and mitochondrial dysfunction in vitro

**Fig. 4:**
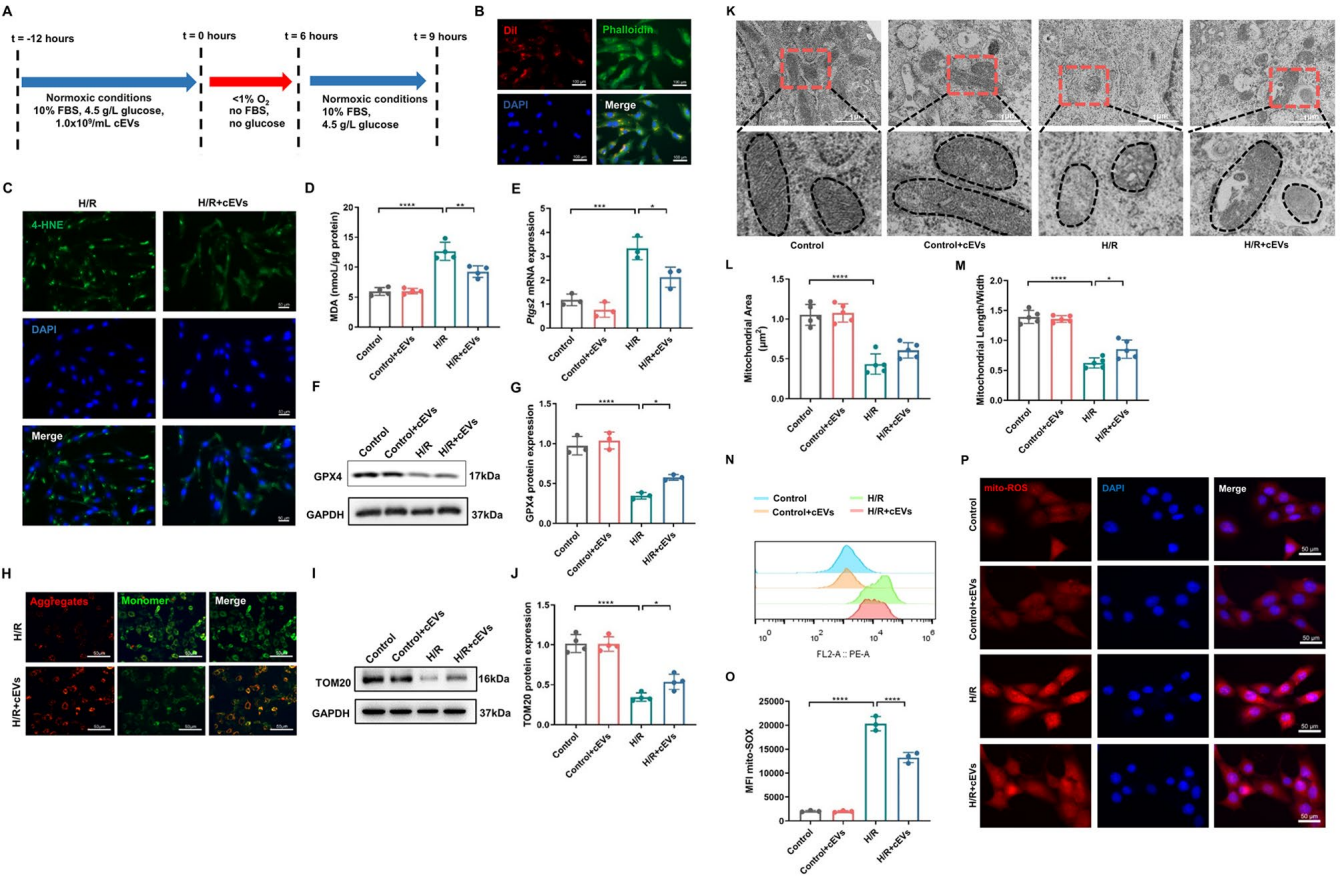
cEVs attenuated oxidative stress-induced cell ferroptosis and mitochondrial dysfunction in vitro. **A** Schematic diagram showing MCM cells under the H/R model and cEVs treatment. **B** Representative micrographs showing cEVs uptaken by MCM cells 6 h after coculture. DiL was used to label cEVs, phallodin was used to label microfilament, DAPI was used to label nuclei (scale bar = 100 μm). **C** Representative micrographs of IF staining showing decreased lipid peroxidation in MCM cells after cEVs treatment (scale bar = 50 μm). **D** MDA production of MCM cells in different groups (*n* = 5). **E** Relative Ptgs2 mRNA expression in MCM cells detected using RT-qPCR (*n* = 3). **F, G** Relative GPX4 protein expression in MCM cells detected using western blot and corresponding quantification analysis (*n* = 3). **H** JC-1 staining measuring the mitochondrial membrane potential of MCM cells. There were increased hyperpolarization (red) and decreased depolarization (green) of mitochondria in MCM cells following cEVs treatment (scale bar = 50 μm). **I, J** Relative Tom20 protein expression in MCM cells detected by western blot and corresponding quantification analysis (*n* = 4). **K-M** Representative TEM images showing the mitochondrial morphology (**K**), mitochondrial area (**L**), and mitochondrial length/width ratio (**M**) in MCM cells detected in TEM images (scale bar = 1 μm, *n* = 5). **N, O** Flow cytometry showing cEVs treatment reduced Mitochondrial ROS content in MCM cells (*n* = 3). **P** Representative micrographs of MitoSOX IF staining showing decreased Mitochondrial ROS content in MCM cells after cEVs treatment (scale bar = 50 μm). (Data were expressed as Mean ± SD. Statistically significant: *p < 0.05, **p < 0.01, ***p < 0.001, ****p < 0.0001)

To further confirm the direct protective role of cEVs on cardiomyocytes, an in vitro ischemia and reperfusion (H/R) cell model was used (Fig. 4A). As shown in Fig. 4B, cEVs can be endocytosed by mouse cardiac myocytes (MCM) 6 h after coculture. Then, we found that cEVs treatment significantly reduced cellular lipid oxidation and deposition compared to the control group (Fig. 4C). Malondialdehyde (MDA), the product of the peroxidation reaction between intracellular oxygen free radicals and unsaturated fatty acids, can effectively reflect the degree of lipid peroxidation in cells and is considered an indicator of cell ferroptosis. Notably, the addition of cEVs significantly reduced H/R-induced MDA production in MCM cells (Fig. 4D). Furthermore, cEVs treatment abolished both the increase of Ptgs2 mRNA expression and the decrease of GPX4 protein expression induced by H/R exposure, as shown in Fig. 4E-G.

Mitochondrial membrane potential (ΔΨm) is a recognized indicator of mitochondrial function [35]. Impaired mitochondrial function leads to the loss of ΔΨm, causing a shift in fluorescence from red (JC-1 aggregates) to green (JC-1 monomers). We detected a significant decrease in ΔΨm in H/R-treated MCM cells. This effect was notably alleviated by cEVs treatment, as evidenced by the presence of more JC-1 aggregates (red) in the H/R + cEVs group compared to the H/R group (Fig. 4H). Additionally, Tom20 protein expression significantly decreased in MCM cells following H/R treatment, and this reduction was remarkably abrogated by cEVs treatment (Fig. 4I, J). Moreover, cEVs treatment mitigated the H/R induced alterations in mitochondrial area and mitochondrial length/width ratio in MCM cells (Fig. 4K-M), which were consistent with in vivo results.

We further analyzed mitochondrial ROS accumulation following H/R treatment by performing MitoSOX staining. Unsurprisingly, H/R treatment induced substantial mitochondrial ROS production in MCM cells, which were significantly suppressed by cEVs treatment (Fig. 4N-P). Taken together, these results indicated that cEVs treatment can reduce the mitochondrial ROS formation, prevent mitochondrial damage induced by H/R, and maintain the mitochondrial homeostasis in cardiomyocytes.

### ATP5a1 was responsible for cEVs-mediated resistance to oxidative stress damage in cardiomyocytes

**Fig. 5:**
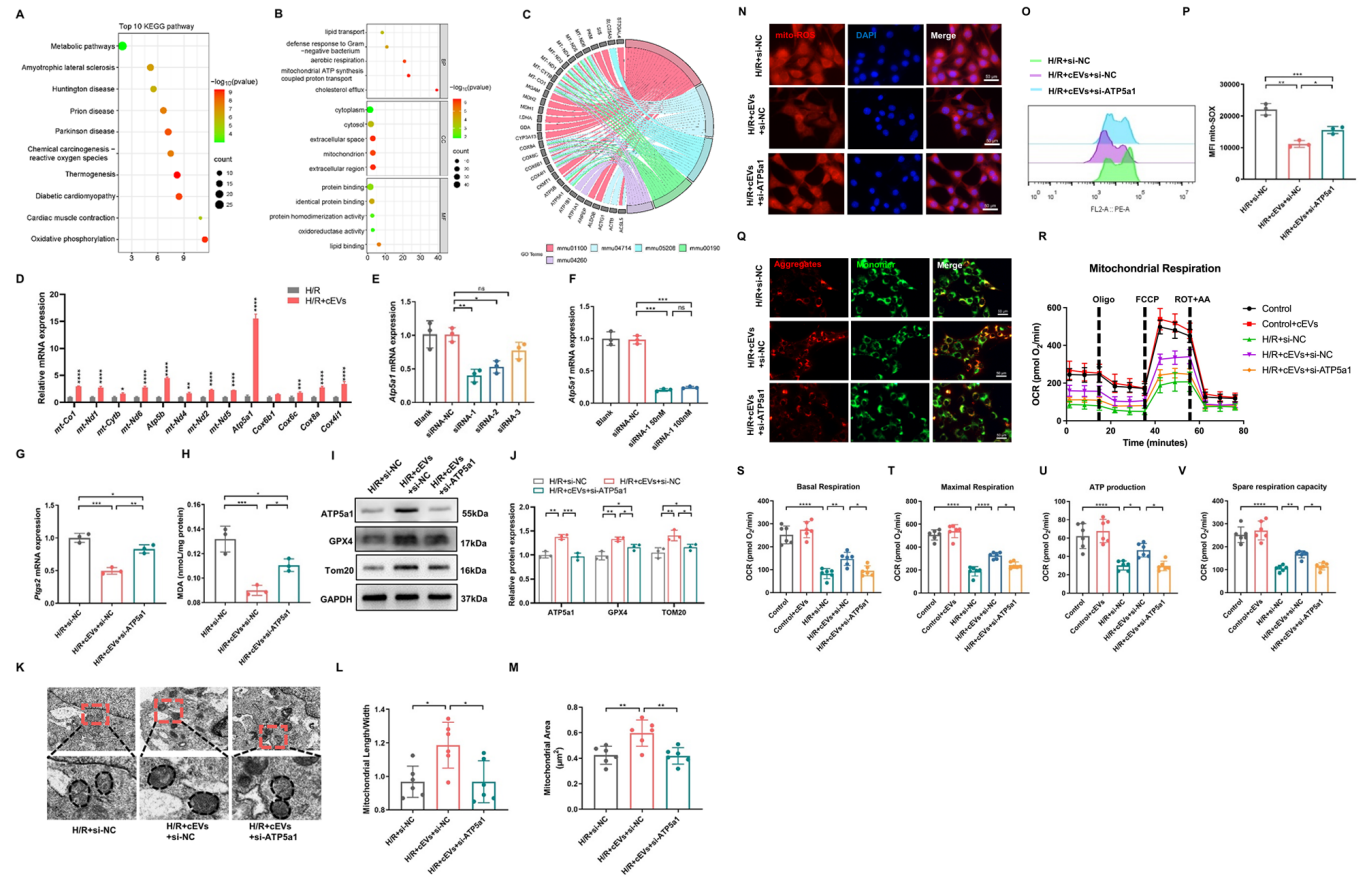
ATP5a1 was responsible for cEVs-mediated resistance to oxidative stress damage in cardiomyocytes. **A** Top 10 of enriched KEGG pathways of top 100 genes from RNA-seq sequencing data of cEVs samples (*n* = 4). **B** Top 5 of GO BP, GO MF, and GO CC analysis of top 100 genes from RNA-seq sequencing data of cEVs samples (*n* = 4). **C** KEGG chord diagram showing genes corresponding to different enrichment KEGG pathways. On the left is the genes, on the right is the mmu term information of the significant enrichment KEGG pathways. **D** Validation of RNA-seq sequencing results of cEVs samples detected by RT-qPCR (*n* = 3). **E** Relative mRNA expression level of ATP5a1 in MCM cells following siRNA-1, siRNA-2, and siRNA-3 transfection (*n* = 3). **F** Relative mRNA expression level of ATP5a1 in MCM cells after transfection of 50nM or 100nM siRNA-1, respectively (*n* = 3). **G** Relative mRNA expression level of Ptgs2 in MCM cells of different groups (*n* = 3). **H** MDA production in MCM cells (*n* = 3). **I, J** Relative protein expression of ATP5a1, GPX4, and Tom20 in different groups detected by western blot and corresponding quantitative analysis (*n* = 3). **K** Representative TEM images showing the mitochondrial morphology of MCM cells in different groups. **L, M** Relative mitochondrial length/width ratio (**L**) and mitochondrial area (**M**) detected in TEM images (scale bar = 1 μm, *n* = 6). **N** Representative micrographs of MitoSOX IF staining showing Mitochondrial ROS content in MCM cells after different treatment (scale bar = 50 μm). **O, P** Flow cytometry showing Mitochondrial ROS content in MCM Cells by MitoSOX and corresponding quantification analysis (*n* = 3). **Q** JC-1 staining showing the mitochondrial membrane potential of MCM cells in different groups (*n* = 5). **R** Mitochondrial oxidative respiration of MCM cells in different groups detected by the cellular OCR. **S**-**V** Basal Respiration (**S**), Maximal Respiration (**T**), ATP production (**U**), and Spare Respiratory Capacity (**V**) of MCM cells in different groups. (Data were expressed as Mean ± SD. Statistically significant: ns: not statistically significant, *p < 0.05, **p < 0.01, ***p < 0.001, ****p < 0.0001)

To address the underlying molecular mechanisms by which cEVs exert their protective function, we extracted RNA form cEVs for RNA-sequencing. The top 100 genes in cEVs were then used for kyoto encyclopedia of genes and genomes (KEGG) pathway and gene ontology (GO) term analysis. As shown in Fig. 5A, the top 10 enriched KEGG pathways mainly include cellular metabolism pathways, thermogenesis, oxidative phosphorylation (OXPHOS), etc. GO analysis suggested that these top 100 genes are related to lipid transport (GO BP), mitochondrial ATP synthesis-coupled proton transport (GO BP), mitochondrial (GO CC), oxidoreductase activity (GO MF), etc. (Fig. 5B). We further analyzed the significantly enriched KEGG pathways involved in cardiac dysfunction and cell ferroptosis, including metabolic pathways (mmu01100), thermogenesis (mmu04714), chemical carcinogenesis-reactive oxygen species (mmu05208), Oxidative phosphorylation (mmu00190), and cardiac muscle contraction (mmu04260). The KEGG enrichment chord diagram (Fig. 5C) illustrated genes corresponding to the aforementioned enriched KEGG pathways. We observed that mitochondrially encoded cytochrome c oxidase I (mt-Co1), mitochondrially encoded NADH dehydrogenase 1 (mt-Nd1), mt-Nd2, mt-Nd4, mt-Nd5, mt-Nd6, mitochondrially encoded cytochrome b (mt-Cytb), cytochrome c oxidase subunit 8 A (Cox8a), Cox6c, Cox6b1, Cox4i1, ATP5b, and ATP5a1 were widely participated in the selected pathways. Therefore, we suspected that cEVs may transport these mRNAs into target cells to reduce mitochondrial damage and cell ferroptosis. Next, we verified these genes by RT-qPCR. As shown in Fig. 5D, compared to H/R control cells, the addition of cEVs significantly increased the expression of above-mentioned genes. Notably, the expression of ATP5a1 was upregulated nearly 15-fold. ATP5a1, a subunit of mitochondrial respiratory chain complex enzyme 5, is closely associated with mitochondrial energy metabolism [36]. Hence, we postulated that ATP5a1 may be responsible for the protective effects of cEVs. We then labeled cEVs with RNA Select green (a nucleic acid stain that selectively labeling RNA in EVs), and cocultured them with MCM cells. The mRNA in cEVs were efficiently internalized and transferred into recipient cells, as indicated by the obvious green fluorescence signal inside these cocultured cells. In contrast, no fluorescence signal was detected in the cells cocultured with the RNA Select alone (Additional file 1: Fig. S3A). To determine whether the ATP5a1 mRNA carried by cEVs is responsible for the increased ATP5a1 expression in target cells, we used Act-D, an inhibitor that blocks endogenous mRNA transcription. Interestingly, cEVs treatment significantly increased ATP5a1 mRNA expression in target cells pretreated with Act-D (Additional file 1: Fig. S3B). We further assessed the protein expression of ATP5a1 in different groups. As shown in Fig. S3C-E (Additional file 1), the ATP5a1 protein was also significantly increased in cardiomyocytes with addition of cEVs compared to the control group.

Next, we synthesized small interfering RNAs (siRNA) of ATP5a1 and found that siRNA-1 exhibited the highest efficiency in inhibiting ATP5a1 expression (Fig. 5E, F). Subsequently, MCM cells were treated with ATP5a1 siRNA and cEVs simultaneously. As expected, cEVs treatment decreased both Ptgs2 expression and MDA production, while the addition of ATP5a1 siRNA abolished the effect (Fig. 5G, H). Moreover, transfection of ATP5a1 siRNA reversed the upregulation of ATP5a1 and GPX4 expression mediated by cEVs in MCM cells subjected to H/R (Fig. 5I, J). We further examined whether transfection of ATP5a1 siRNA could also abrogate the mitochondrial homeostasis maintained by cEVs. Unsurprisingly, inhibition of ATP5a1 by siRNA transfection obviously blocked cEVs mediated recovery in Tom20 protein expression (Fig. 5J) and mitochondrial morphology injury (Fig. 5K-M). Additionally, the reduction of mitochondrial ROS induced by cEVs in MCM cells was also counteracted by the addition of ATP5a1 siRNA (Fig. 5N-P). Moreover, JC-1 staining showed that ATP5a1 siRNA transfection blocked cEVs mediated repair of ΔΨm in MCM cells, as indicated by decreased JC-1 aggregates (red) in the H/R + cEVs + si-ATP5a1 group compared to that in the H/R + cEVs + si-NC group (Fig. 5Q). The generation of ATP through OXPHOS is an essential role of mitochondria in mammalian cells [37]. To better detect mitochondria function of MCM cells, we performed O_2_ consumption rate (OCR) analysis. As shown in Fig. 5R-V, H/R treatment notably impaired the overall mitochondrial respiratory capacity of MCM cells, resulting in reduced levels of basal respiration, maximal respiration, ATP production, and spare respiratory capacity. In contrast, the decrease in these oxidative phosphorylation (OXPHOS) parameters in MCM cells were reversed by cEVs treatment, but such improvements in mitochondrial OXPHOS were abolished by the simultaneous transfection of si-ATP5a1. Thus, these findings provided compelling evidence that cEVs can protect cardiomyocytes from oxidative stress-induced mitochondrial damage and ferroptosis through the transport of ATP5a1.

### cEVs alleviated H/R-induced ferroptosis and mitochondrial damage in NMCMs by transporting ATP5a1

**Fig. 6:**
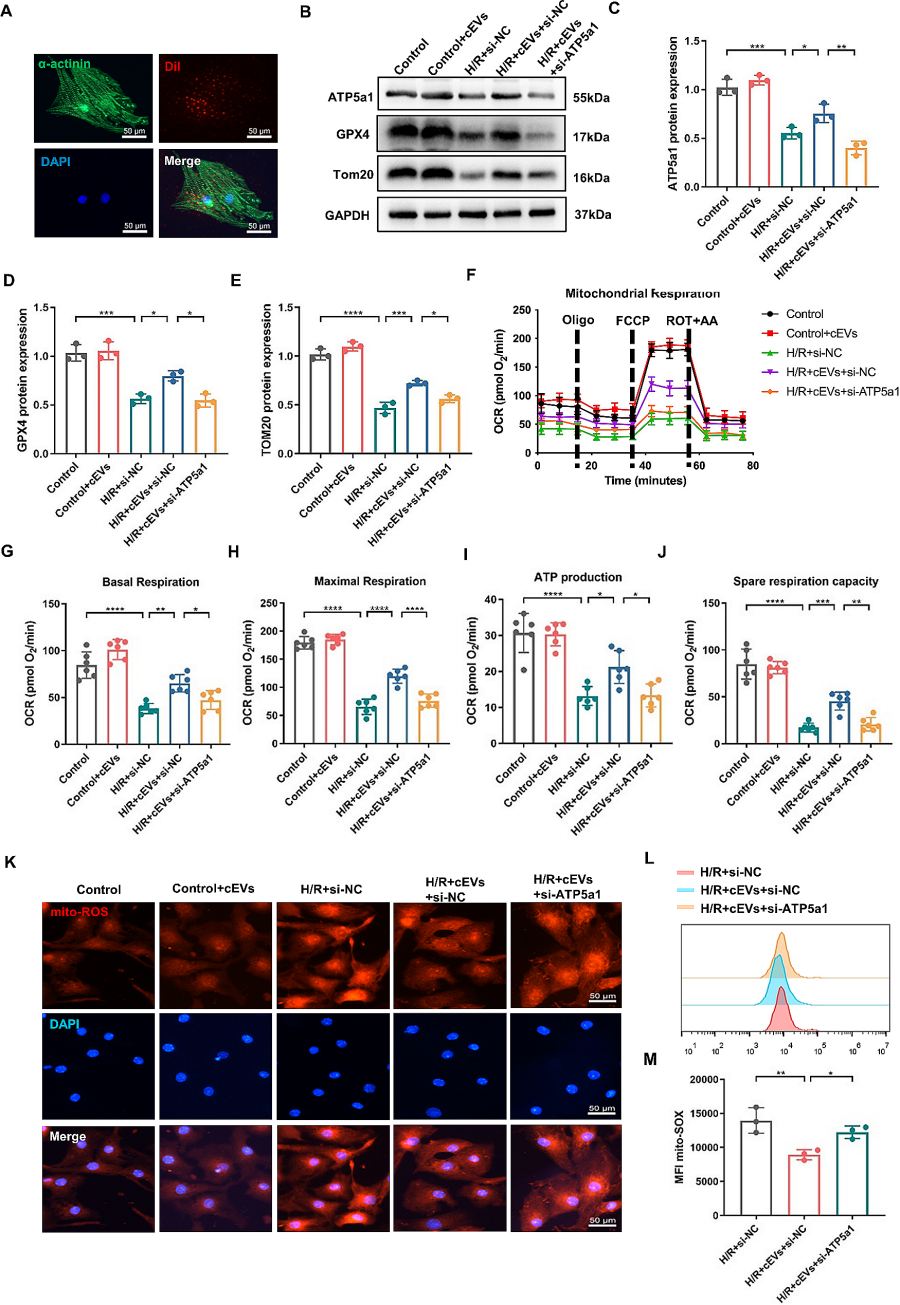
cEVs alleviated H/R induced ferroptosis and mitochondrial damage in NMCMs by transporting ATP5a1. **A** Representative micrograph showing cEVs uptaken by isolated NMCMs. **B** Relative protein expression of ATP5a1, GPX4, and Tom20 of NMCMs in different groups detected by western blot (*n* = 3). **C-E** Quantitative analysis of protein expression of (**C**) ATP5a1, (**D**) GPX4, and (**E**) Tom20 of NMCMs in different groups (*n* = 3). **F** Mitochondrial oxidative respiration of NMCMs in different groups detected by the cellular OCR (*n* = 6). **G-J** Basal Respiration (**G**), Maximal Respiration (**H**), ATP production (**I**), and Spare Respiratory Capacity (**J**) of NMCMs in different groups. **K** Representative micrographs of MitoSOX IF staining showing Mitochondrial ROS content in NMCMs (scale bar = 50 μm). **L, M** Flow cytometry detection showing Mitochondrial ROS content in NMCMs revealed by MitoSOX staining and corresponding quantification analysis (*n* = 3). (Data were expressed as Mean ± SD. Statistically significant: *p < 0.05, **p < 0.01, ***p < 0.001, ****p < 0.0001)

We then isolated neonatal mouse cardiomyocytes (NMCMs) to verify the above findings. Figure 6A revealed that cEVs can also be endocytosed by NMCMs. Additionally, we observed an obvious decrease in protein expression of ATP5a1, GPX4, and Tom20 in NMCMs subjected to H/R treatment (Fig. 6B-E). OXPHOS parameters in NMCMs, including basal respiration, maximal respiration, ATP production, and spare capacity, also decreased after H/R stimulation. However, these alterations induced by H/R were notably alleviated by cEVs treatment (Fig. 6F-J). Interestingly, we found that simultaneous transfection of si-ATP5a1 significantly counteracted the protective effect of cEVs on NMCMs. Subsequently, we measured the mitochondrial ROS in NMCMs by using MitoSOX staining. Figure 6K-M showed a large amount of mitochondrial ROS production in NMCMs following H/R treatment. In contrast, cEVs treatment significantly reduced the accumulation of mitochondrial ROS in NMCMs. As expected, transfection of si-ATP5a1 showed a reversed effect, as the amount of mitochondrial ROS was increased in the H/R + cEVs + si-ATP5a1 group compared to the H/R + cEVs + si-NC group.

### ATP5a1 in cEVs mainly derived from cardiomyocytes in mice heart

The heart consists of different types of cells, mainly including cardiomyocytes, endothelial cells, fibroblasts, and macrophages. To figure out the cellular origin of ATP5A1 in cEVs, we co-stained ATP5a1 and markers of different cells. Results in Fig. S4A showed that almost all the fluorescent signal of ATP5a1 overlapped with α-actinin, but not CD31, Vimentin, or CD45. These results indicated that ATP5a1 is mostly expressed in cardiomyocytes. Then, we separated cardiomyocytes and non-cardiomyocytes components from the mouse heart. As shown in Fig. S4B (Additional file 1), Tnnt2 expression in cardiomyocytes group was much higher than that in the non-cardiomyocytes group, indicating a successful separation between cardiomyocytes and non-cardiomyocytes. Moreover, ATP5a1 expression was also highly expressed in cardiomyocytes group compared to non-cardiomyocytes group (Additional file 1: Fig. S4B), suggesting that ATP5a1 is mainly expressed in cardiomyocytes. Since cEVs are derived from heart tissue, we intend to explore which cell population is the major source of cEVs production. To this end, we extracted RNA from cEVs and examined the marker abundance of different cell types. Obviously, the expressions of Tnnt2 and myosin heavy chain 6 (Myh6), markers of cardiomyocytes, were much higher than those of other cell types, including Pecam1, Kdr, Vimentin, α-SMA, and mAdgre1 (Additional file 1: Fig. S4C). Collectively, these results suggested that the ATP5a1 in cEVs is mainly derived from cardiomyocytes in the heart and cEVs are mainly composed of EVs secreted by cardiomyocytes.

### ATP5a1 overexpression improved the therapeutic potential of ADSC-EVs in mice MI/R models

**Fig. 7:**
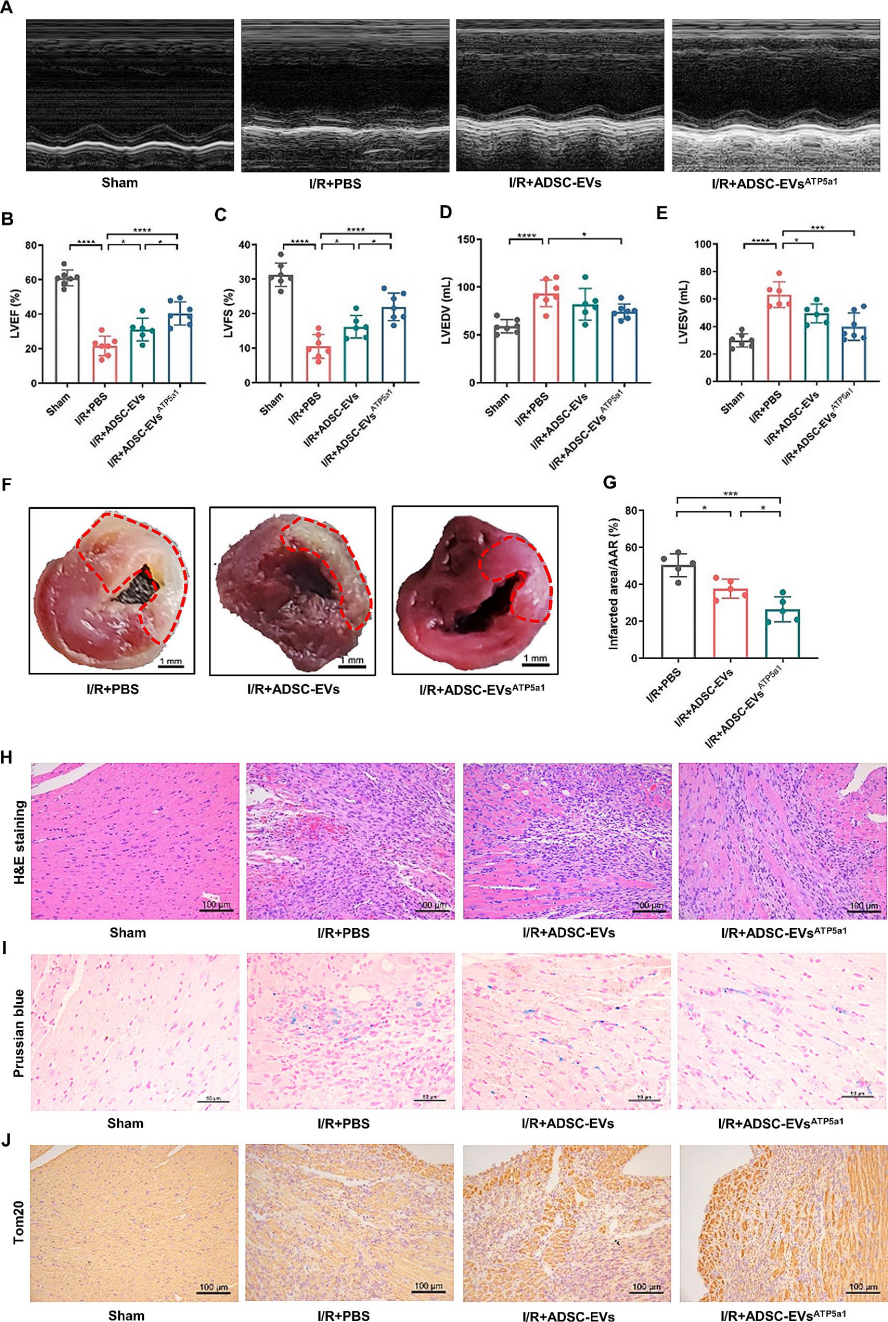
Overexpression of ATP5a1in ADSC improved the therapeutic efficacy of ADSC-EVs in murine MI/R models. **A** Representative image of cardiac function of mice treated with PBS, ADSC-EVs, or ADSC-EVs^ATP5a1^ determined by M-mode echocardiography at day 3 post MI/R operation (*n* = 6–7). **B-E** Results of LVEF (**B**), LVFS (**C**), LVEDV (**D**), and LVESV (**E**) in mice detected by echocardiogram (*n* = 6–7). **F, G** Representative TTC staining and corresponding quantification analysis showing infarcted area/AAR ratio of mice heart in different groups (scale bar = 1 mm, *n* = 5). **H** Representative H&E staining of mice heart in different groups (scale bar = 100 μm, *n* = 6). **I** Representative micrographs of Prussian blue staining showing decreased iron accumulation in mice heart after ADSC-EVs^ATP5a1^ treatment (scale bar = 50 μm, *n* = 6). **J** Representative micrographs of Tom20 IHC staining showing alleviated mitochondrial damage in mice heart after ADSC-EVs^ATP5a1^ treatment (scale bar = 100 μm, *n* = 6). (Data were expressed as Mean ± SD. Statistically significant: *p < 0.05, **p < 0.01, ***p < 0.001, ****p < 0.0001)

Next, to test whether exogenous over expression of ATP5a1 in EVs could protect the heart from MI/R injury, we treated mice with ADSC-derived EVs overexpressing ATP5a1(ADSC-EVs^ATP5a1^) (Additional file 1: Fig. S5A). After characterization of ADSC by morphological (Additional file 1: Fig. S5B) and flow cytometry analysis (Additional file 1: Fig. S5C), we transfected ADSC with the ATP5a1 plasmid. Subsequently, EVs were collected from the culture media of ADSC exhibiting high expression of ATP5a1. As shown in Fig. S5D-F (Additional file 1), transfection of ATP5a1 plasmid significantly upregulated the mRNA and protein expression of ATP5a1 in ADSC compared to the plasmid control group. Then, the isolated ADSC-EVs was further characterized by western blot, TEM, and NTA analysis (Additional file 1: Fig. S5G-I). RT-qPCR analysis determined that ATP5a1 was highly expressed in EVs derived from ADSC pretreated with ATP5a1 plasmid (Additional file 1: Fig. S5J). Further, ADSC-EVs or ADSC-EVs^ATP5a1^ were intramyocardial injected into mice heart post MI/R injury to assess their therapeutic effects. Figure 7A-E showed that mice receiving ADSC-EVs treatment exhibited increased LVEF and LVFS, as well as decreased LVEDV and LVESV compared to mice treated with PBS. These effects were further improved by the administration of ADSC-EVs^ATP5a1^. Additionally, compared with the PBS group and ADSC-EVs group, mice in the ADSC-EVs^ATP5a1^ group presented a significant reduction in myocardial infarct size (Fig. 7F, G) and inflammatory infiltration (Fig. 7H). Besides, IHC staining suggested that, compared to the PBS group, ADSC-EVs treatment significantly inhibited the accumulation of iron (Fig. 7I) and increased the protein expression of Tom20 (Fig. 7J) in the heart, which was further improved in the ADSC-EVs^ATP5a1^ group. Collectively, these results suggested that exogenous overexpression of ATP5a1 in EVs represents a potential therapeutic strategy for MI/R injury.

## Discussion

In our current study, we found a significant inhibition of cardiomyocytes ferroptosis and a reduction in cardiac injury in murine MI/R injury models following the intramyocardial administration of cEVs. Additional evidence revealed that cEVs can preserve mitochondrial homeostasis by delivering ATP5a1 into cardiomyocytes. More importantly, for translational practice, we demonstrated that EVs derived from ADSC with ATP5a1 overexpression exhibited much better therapeutic effect on MI/R injury. Our findings highlighted the protective role of cEVs in ischemia-reperfusion heart injury. Moreover, ATP5a1 may represent an important therapeutic target to minimize myocardial damage induced by MI/R injury.

Loss of cardiomyocytes is a main pathological process that contributes to cardiac dysfunction post MI/R injury. Among other processes, apoptosis is certified as an important form that causes a decrease in cardiomyocytes [38, 39]. However, on the 3rd day after MI/R surgery, we found that the proportion of cardiomyocytes apoptosis in cEVs-treated mice showed no significant difference than that in the PBS treatment group. Instead, the ferroptosis of cardiomyocytes decreased significantly after cEVs treatment. This may be explained by a recently published study in which the authors found that apoptosis may be involved in the acute phase of cell damage caused by MI/R injury and returns to normal after 24 h of reperfusion, whereas ferroptosis occurs later than apoptosis [40]. In the present study, a large accumulation of iron ion, deposition of lipid peroxide, and increased MDA production all suggested that cardiomyocyte ferroptosis occurred on day 3 after MI/R. Importantly, cEVs treatment dramatically inhibited cardiomyocyte ferroptosis. In vitro results further confirmed that the addition of cEVs significantly alleviated cardiomyocyte ferroptosis induced by H/R treatment. Therefore, intramyocardial injection of cEVs may improve cardiac function after MI/R injury by reducing ferroptosis in cardiomyocytes.

Previous studies showed that mitochondria protective strategies could prevent myocardial injury by inhibiting cardiomyocytes ferroptosis [41, 42]. In this study, we found that cardiomyocytes ferroptosis was accompanied by obvious mitochondrial damage. The addition of cEVs not only alleviated myocardial mitochondrial damage but also suppressed the cardiomyocytes ferroptosis. Moreover, cEVs treatment significantly improved the function of mitochondria in cardiomyocytes. Mito-ROS production, which may lead to mitochondrial dysfunction and ultimately cellular ferroptosis [43], was also reduced after cEVs treatment. These results indicated that cEVs possess the capability to attenuate MI/R injury-induced cardiomyocytes ferroptosis by suppressing Mito-ROS generation and preserving mitochondrial homeostasis.

A substantial body of evidence has revealed that EVs can transfer their RNA cargos to recipient cells, serving as crucial mediators of intercellular communication [44, 45]. Other reports have demonstrated that EVs from various cell types can hinder mitochondrial damage in target cells by stabilizing mitochondrial DNA, transporting molecules related to mitochondrial respiratory chain, mitochondrial cleavage/fusion molecules, or intact mitochondria. Consequently, these actions contribute to the restoration of cellular energy metabolism and the reduction of tissue ischemia injury [27, 28]. Here, we found that ATP5a1, a subunit of mitochondrial respiratory chain complex enzyme 5 [36], was enriched in cEVs. Studies have demonstrated that ATP5a1 is implicated in multiple diseases, and the modulation of ATP5a1 could alleviate tissue ischemia injury by maintaining mitochondrial energy metabolism and preventing mitochondrial damage. For example, it was reported that ATP5a1 expression significantly decreased in Leydig cells after chronic stress, and the transfection of ATP5a1 siRNAs inhibited testosterone synthesis in Leydig cells by inducing mitochondrial damage [46]. Jonckheere et al. found that a defect in ATP5a1 causes fatal neonatal mitochondrial encephalopathy [47]. Another report revealed that ATP5a1 overexpression decreased mitochondrial damage, attenuated cytotoxicity, restored ATP levels, and increased neuronal survival in a mouse model of poly (GR)-induced neurotoxicity [48]. The current study demonstrated notable damage to mitochondrial structure and function, along with reduction of ATP5a1 expression in cardiomyocytes both in vitro and in vivo. Moreover, cEVs can transfer ATP5a1 into cardiomyocytes and alleviate mitochondrial damage. These results highlighted the vital role of ATP5a1 carried by cEVs in maintaining cardiomyocytes mitochondrial homeostasis post MI/R injury. More importantly, the regulation of ATP5a1 may represent a promising strategy for treating ischemia diseases related to mitochondrial damage.

Many studies have confirmed the efficacy of EVs from stem cells or other cultured cells in treating various diseases [49, 50]. Several recent studies [51–53], including ours [29], have highlighted the role of tissue-derived EVs in the pathogenesis of diseases. In the present study, we explored, for the first time, the efficacy of normal heart tissue-derived EVs in treating ischemia heart disease. Our in vivo results demonstrated the obvious efficacy of cEVs through intramyocardial injection in murine MI/R injury models. This efficacy included the improvement of cardiac function, reduction of infarct size, maintenance of mitochondria function, and inhibition of ferroptosis in cardiomyocytes. The marked efficacy of cEVs may attributed to their tissue specificity. Additionally, as EVs derived from tissue can accurately reflect the normal microenvironment of that tissue or organ, they may be more efficient in reversing abnormal microenvironments in pathological conditions. Therefore, further studies are still needed to explore whether EVs derived from different sources exhibit specific efficacy in their respective parent tissue. On the other hand, it must be noted that the heart consists of different cell types, each capable of secreting EVs. Our results showed that the majority of cEVs were secreted by cardiomyocytes. This can be attributed to the proportion and relatively large morphology of cardiomyocytes in the whole heart compared to other cell types. In addition, we found that ATP5a1 is primarily expressed in cardiomyocytes and is hardly detected in other types of heart cells. This phenomenon may be explained by the involvement of ATP5a1 in cell OXPHOS, and since cardiomyocytes contain a large number of mitochondria with a higher demand for energy metabolism. However, how ATP5a1 improves mitochondrial function at the molecular level and the mechanisms by which cardiomyocytes selectively load ATP5a1 into EVs in cardiac homeostasis requires further investigation.

Previous studies have shown that adenoviral-mediated overexpression of ATP5a1 could prevent cardiomyocyte damage and attenuate myocardial dysfunction under an endotoxemic model or oxidative stress-induced myocardial injury [54, 55]. However, the potential recombinant risk of viral vectors remains [56]. In contrast, EVs possess the capability to cross physical barriers, intrinsic stability in circulation, inherent targeting properties, and decreased immune clearance, all of which favor their utilization as drug delivery applications for the treatment of various diseases [57, 58]. On the other hand, while tissue-derived EVs demonstrate remarkable efficacy, acquiring normal heart tissue for EVs isolation remains challenging. For clinical translational practice, in this study, ADSC was chosen as the cellular type to produce therapeutic EVs due to their convenient source, superior abundance, and gene modifiability [59, 60]. We overexpressed ATP5a1 in ADSC, isolated the ADSC-derived EVs, and confirmed their much better therapeutic effect on MI/R injury than their normal counterparts. These results suggested that once promising therapeutic molecules in EVs derived from normal tissue, especially vital organs, have been identified, other well-established and efficient strategies can be employed to transport such functional molecules to the injured area for therapeutic purposes. This not only avoids dependence on normal tissue to obtain the therapeutic EVs but also sheds new light on EVs-mediated therapy in the repair of tissue damage.

## Conclusions

In summary, the present study demonstrated the cardio-protective roles of cEVs in suppressing cardiomyocytes ferroptosis post MI/R injury by delivering ATP5a1. Importantly, the modulation of ATP5a1 may be an effective strategy to alleviate myocardial injury and heart dysfunction in ischemia heart disease.

## Methods and materials

### Mice

C57BL/6 N mice (male, 6–8 weeks old) were supplied by Charles River Laboratory Animal co., Ltd. (Zhejiang, China). Mice were housed in the specific-pathogen-free (SPF) room with temperature-controlled (23–24 °C) and 12 h light and dark cycle. All the mice had free access to food and water. All animal procedures are conducted in compliance with the Guide for the Care and Use of Laboratory Animals published by the National Institutes of Health (NIH Publication No. 85 − 23, 1996, revised 2011) and approved by the Institutional Animal Care and Use Committee of Tongji University (Number: TJBB00122103).

### Establishment of murine MI/R injury models

Mice MI/R injury models were set up as previously described [61]. Briefly, 6–8 weeks old male mice were anesthetized by 1% isoflurane with respiration controlled by a rodent ventilator (Nemi Scientific, Inc., Framingham, MA). Subsequently, a left thoracotomy was performed at the fourth intercostal space to exposure the heart. Left anterior descending coronary artery (LAD) was ligated with an 8 − 0 monofilament nylon suture. After 45 min, reperfusion was generated by releasing the ligation. Immediately, cEVs or ADSC-derived EVs (2.0 × 10^9^ particles/uL in 50 µL PBS) were intramyocardial injected into the peri-infarct myocardial region at 2 different points (*n* = 6–8 per group). Mice in control group only received 50 µL PBS intramyocardial injection. Sham-operated mice underwent the same procedure without LAD ligation (*n* = 6–8). At the indicated time points, mice were sacrificed by anesthesia, and tissue were subsequently harvested for histological analysis. In addition, the ratio of heart weight (HW) to body weight (BW) and HW to tibia length (TL) in each group were also analyzed.

### Cell culture

Mouse cardiac myocytes (MCM) cells were purchased from the American Type Culture Collection (ATCC, Gaithersburg, MD, USA). To simulate MI/R injury in vitro, MCM cells were exposed to hypoxia-reoxygenation (H/R) treatment. In brief, ischemia was simulated by culturing cells with Dulbecco’s modified Eagle’s medium (DMEM) containing no glucose and free of FBS in a sealed bag maintaining O_2_ < 1% for 6 h at 37 °C. For reperfusion, the medium was changed to fresh DMEM containing high glucose and 10% FBS. Cells were then cultured at 21% O_2_ for another 3 h. To test the protective effect of cEVs, MCM cells were pretreated with 1 × 10^9^ particles/mL cEVs for 12 h before ischemia treatment.

### EV isolation

cEVs were isolated from 6 to 8 weeks old male mice heart as previously described [29]. After perfusing with PBS, heart tissue was digested in 0.1% type II collagenase (Sigma-Aldrich, USA) at 37 °C for 30 min. Subsequently, the digested tissue was centrifuged at 300 g for 5 min, 2000 g for 10 min, and 10,000 g for 10 min to remove cells and debris. Then, the supernatant was collected and ultracentrifuged at 120,000 g for 2 h, twice (Optima L-100XP Ultracentrifuge, Beckman Coulter). Finally, the pellet was resuspended in PBS and stored in -80 °C for further use. For ADSC derived EVs, the cells were cultured with DMEM consisting of 10% FBS (with EV-depleted using ultracentrifugation at 120,000 g for 18 h at 4 °C), 1% PS and 2 mM L-glutamine culture media for 48 h. Then, the cell culture media were collected and centrifuged at 2,000 g for 30 min, 10,000 g for 30 min, 120,000 g for 70 min twice at 4 °C. Then, the EVs was suspended in pre-cooled PBS and stored at -80 °C for further use.

### Malondialdehyde (MDA) detection

The relative MDA concentration in cell lysates was determined by the level of lipid peroxidation using a detection Kit (Beyotime Biotechnology, China) according to the manufacturer’s instructions. Briefly, cells were washed with PBS and lysed with lysis buffer. Then, the supernatant was collected for MDA detection after centrifugation. Finally, a spectrophotometer reader was used to measure the absorbance of cellular MDA at 532 nm. Protein concentrations were determined by BCA protein assay kit (Thermo Fisher Scientific, USA) to normalize MDA concentration in each group.

### MitoSOX assay

Mitochondrial reactive oxygen species (ROS) was detected by MitoSOX Red (Yeason, China) according to manufacturer’s protocols. Briefly, cells were seeded in 24-well plates. After treatment with H/R ± EVs (1.0 × 10^9^ particles/mL), cells were added with 5 µM MitoSOX and cultured at 37 °C for 10 min in the dark. After collecting and washing twice with HBSS buffer containing Ca^2+^ and Mg^2+^, cells were re-suspended in 200 µL PBS and red fluorescence was detected by flow cytometry analysis (excitation 488 nm, emission 690/50 nm). Analysis of ROS levels in vivo was performed according to previous research [62]. In brief, hearts in different group were perfused with ice-cold PBS and removed quickly. Then, ventricular tissue near the apex of the heart was separated and embedded in OCT and snap-frozen in liquid nitrogen for 15 sec. After embedding, 5-µm-thick sections were stained with dihydroethidium (DHE, Invitrogen) at 37 °C for 10 min, followed by DAPI staining. Pictures were captured using a fluorescence microscope.

### Transfection of si-RNA

When cells reached around 60–70% confluence, they were transfected with 50 or 100 nmol/L of ATP5a1 siRNA (GenePharma, China) or negative control through Lipofectamine 3000 (Life Technologies) in Opti-MEM medium following manufacturer’s protocols. 24 h after transfection, cells were treated with cEVs (1.0 × 10^9^ particles/mL) as described above and collected for further experiments.

### Plasmid transfection

ATP5a1 plasmid or empty vector (Hanbio Biotechnology Co., Ltd. China) was transfected into ADSC when cells reached 60–70% confluence using Lipofectamine 3000 (Life Technologies) according to manufacturer’s introductions. After 48 h of transfection, ADSC derived EVs (ADSC-EVs) were isolated and purified from the culture media of ADSC.

### RNA library preparation and sequencing

cEVs from 4 mice were isolated for RNA sequencing. Total RNA was extracted from cEVs by Trizol Reagent (Invitrogen, CA, USA). The concentration and purity of RNA samples were determined by a NanoDrop 2000 spectrophotometer (Thermo). The RNA libraries were constructed using the TruSeq Stranded Total RNA Library Prep Kit (Illumina) and sequenced using a HiSeq 4000 sequencing system (Illumina). The sequencing service was provided by CloudSeq Biotech (Shanghai, China). Gene Ontology (GO) and Genomes (KEGG) pathway enrichment analyses were performed based on the top 100 expressed genes in sequencing results to explore the significant pathways.

### Echocardiography analysis

3 days and 28 days after MI/R injury operation, a Vevo2100 Ultrasound system (Visual Sonics, Canada) was applied to assess murine cardiac function, including the left ventricular ejection fraction (LVEF), left ventricular fraction shortening (LVFS), left ventricular end-diastolic volume (LVEDV), and left ventricular end-systolic volume (LVESV) in two-dimensional long axis views.

### Histological examination

The mouse heart tissue were fixed with 4% paraformaldehyde for 48 h and embedded in paraffin. Subsequently, the heart tissue were sliced into 5-µm sections. H&E staining was used to determine pathological changes of myocardium tissue, whereas Masson trichrome staining was used to detect cardiac fibrosis. Wheat germ agglutinin (WGA) staining was applied for analysis of myocyte cross-sectional area. Terminal deoxynucleotidyl transferase dUTP nick end labelling (TUNEL) staining was used to detect myocardial apoptosis. Triphenyltetrazolium chloride (TTC) staining was used to detect infarcted area. These data were measured and analyzed by Image J software (National Institutes of Health, USA).

### Real-time quantitative polymerase chain reaction (RT-qPCR) analysis

Total RNA was extracted using Trizol reagent (Invitrogen), followed by cDNA synthesis with a Prime Script RT reagent kit (Takara Bio, Inc., Otsu, Japan). Then, qPCR was performed using SYBR Green MasterMix (Applied Biosystems; Thermo Fisher Scientific, Inc.) with appropriate primer sequences (Table S1). Measurements were normalized to the expression of β-actin mRNA. The 2^–∆∆CT^ or 2^–∆CT^ method was used to calculate relative expression of different genes.

### Western blot analysis

Proteins were extracted in RIPA buffer containing protease and phosphate inhibitor, followed by concentration detection using Bicinchoninic acid assay (BCA) protein estimation kit (Thermo Fisher Scientific, USA) in accordance with the manufacturer’s instruction. Total proteins were separated via sodium dodecyl sulfate-polyacrylamide gel (SDS-PAGE), and transferred to polyvinylidene difluoride (PVDF) membranes. Consequently, the PVDF membrane was blocked with 5% nonfat milk for 1 h at room temperature, and then incubated with a primary antibody CD9 (Abcam, UK), Alix (Abcam, UK), TSG101 (Abcam, UK), Calnexin (Abcam, UK), ATP5a1 (Abclonal, China), GPX4 (Abclonal, China), Tom20 (Abclonal, China) and GAPDH (Santa Cruz Biotechnology, USA). Then, the membrane was incubated with an anti-mouse HRP (Cell Signaling Technology, USA) or anti-rabbit HRP secondary antibody (Cell Signaling Technology, USA). The amount of protein expression was visualized with ECL reagent and quantified using Image J software.

### Statistical analysis

Results were presented as mean ± SD. Comparisons between two groups were made using Student’s t-test, whereas the data obtained from multiple groups were compared using one-way analysis of variance (ANOVA), followed by Tukey’s multiple comparisons test. A *P*-value less than 0.05 was considered significant. GraphPad Prism 9.0 (Graph Pad Prism Software Inc., San Diego, CA, USA) were used for statistical analysis.

### Electronic supplementary material

Below is the link to the electronic supplementary material.

Supplementary Material 1

Supplementary Material 2

## Data Availability

The datasets used and analysed during the current study are available from the corresponding author on reasonable request.

## Abbreviations

EVs: Extracellular vesicles
cEVs: Heart derived EVs
MI/R: Myocardial ischemia-reperfusion
ADSC: Adipose-derived stem cells
4-HNE: 4-Hydroxynonenal
MDA: Malondialdehyde
H/R: Hypoxia-reoxygeneration
OXPHOS: Oxidative phosphorylation
NMCMs: Neonatal mouse cardiomyocytes
OCR: Oxygen consumption rate
