# Supplementary material for: Cardiac-derived extracellular vesicles improve mitochondrial function to protect the heart against ischemia/reperfusion injury by delivering ATP5a1
Source: J Nanobiotechnology. 2024 Jul 1;22:385. doi: 10.1186/s12951-024-02618-x (PMC11218245; doi:10.1186/s12951-024-02618-x)
Supplement: Supplementary file 2 — Supplementary Material 2 [file 12951_2024_2618_MOESM2_ESM.docx]

**Supporting information**

**Cardiac-derived extracellular vesicles improve mitochondrial function to protect the heart against ischemia/reperfusion injury by delivering ATP5a1**

Xuan Liu^1-3^, Qingshu Meng ^1-2^, Shanshan Shi ^1-2^, Xuedi Geng^1-2^, Enhao Wang^1-2^, Yinzhen Li^1-2^, Fang Lin^1-2^, Xiaoting Liang^1-2^, Xiaoling Xi^4^, Wei Han^4^, Huimin Fan^1-3^*, Xiaohui Zhou^1-2^*

1. Research Center for Translational Medicine, Shanghai East Hospital, Tongji University School of Medicine, Shanghai 200092, China.

2. Shanghai Heart Failure Research Center, Shanghai East Hospital, Tongji University School of Medicine, Shanghai 200092, China.

3. Department of Cardiothoracic Surgery, Shanghai East Hospital, Tongji University School of Medicine, Shanghai 200092, China.

4. Department of Heart Failure, Shanghai East Hospital, Tongji University School of Medicine, Shanghai 200092, China

*Corresponding Author:

Xiaohui Zhou, M.D., Ph.D.

E-mail: zxh100@tongji.edu.cn

or

Huimin Fan, M.D., Ph.D.

E-mail: [frankfan@tongji.edu.cn](mailto:frankfan@tongji.edu.cn)

**Supplemental Methods and Materials**

**Neonatal mouse cardiomyocytes (NMCMs) culture and treatment**

NMCMs were isolated and cultured using a neonatal heart dissociation kit (MACS, NO. 130-098-373) according to manufacturer’s protocols. Briefly, the NMCMs were prepared by enzymatic digestion of hearts obtained from newborn (1~3 days old) mouse and plated on a 6-well plate (precoated with 1% Laminin) at a density of 5 × 10^5^ cells/cm^2^ in DMEM/F12 medium and maintained at 37°C with 5% CO_2_. To mimic the ischemia/reperfusion condition in vivo, NMCMs were treated with hypoxia-reoxygenation (H/R) (6 h of hypoxia followed by 3 h of re-oxygenation). NMCMs was pretreated with cEVs (1.0 x 10^9^ particles/mL) for 12 h before H/R.

**Isolation of mouse cardiomyocytes and non-myocyte cardiac populations**

Cardiac myocytes and non-myocyte cardiac populations were isolated as previously described (1). In brief, mice at 6-8 weeks were anesthetized. Then, heart was immediately flushed by injection of EDTA buffer into the right ventricle, with digestion achieved by sequential injection of 10 mL EDTA buffer, 3 mL perfusion buffer, and 30 mL collagenase buffer into the left ventricle (LV). Constituent chambers (atria, LV, and right ventricle) were then gently separated into 1-mm pieces. Thereafter, cell suspension was passed through a 100-μm filter, followed by 3 sequential rounds of gravity settling. The cell pellet in each round was enriched with pure cardiomyocytes fraction, whereas the supernatant from each round contained the non-myocyte populations.

**Culture of adipose derived stem cells (ADSC)**

ADSC were isolated and cultured from the groin adipose tissue of mice (3-4 weeks) as previously reported (2). Adipose tissues were cut into pieces and incubated with 0.1% collagenase I (Thermo Fisher Scientific, USA) at 37°C for 30 min. Then, the mixture was centrifuged at 2000 rpm for 10 min. The cells were suspended in DMEM consisting of 10% FBS, 1% Penicillin-Streptomycin (PS) and 2 mM L-glutamine and incubated at 37℃ in a humidified atmosphere with 5% CO_2_. ADSC with 3~5 passages were used in the in vivo experiment. The identification of ADSC was carried out by flow cytometry analysis (BD Bioscience, USA) with positive cell surface markers, including CD 45, Scar-1 and negative markers, such as CD 31 and CD 34 (BioLegend, USA).

**Nanoparticle tracking analysis (NTA)**

The cEVs were evaluated with ZetaView NTA technique by Particle Metrix (Meerbusch, Germany) following manufacturer’s protocols. Briefly, cEVs were diluted at 1:1000 to 1:2500 with PBS. The light scattered by the cEVs and moving of cEVs under Brownian motion was recorded with optimized setting. NTA 3.1 software was used for analyzing particle size distribution.

**Transmission electron microscopy (TEM)**

The fresh-prepared cEVs were loaded on the grid and incubate with 2% phosphotungstic acid solution for 1 -10 min. After removing the excess solution, transmission electron microscope (TEM) images were immediately obtained for further analysis (Hitachi, HT7700).

**Biodistribution detection**

To detect the biodistribution of cEVs in different organs in vivo, mice were intramyocardially injected with DiR-labeled cEVs (Thermo Scientific, USA). At 24 h, 48 h, and 72 h post injection, heart, liver, spleen, lung, kidney, and brain were harvested for ex vivo fluorescence imaging using IVIS imaging system (Xenogen, USA). In addition, to confirm that cEVs can be endocytosed by cardiomyocytes in vivo, cEVs were stained with Dil in vitro and then injected into the heart. 24 h, 48 h, and 72 h after the operation, the mice hearts were collected and cut into 6 μm sections for immunofluorescence staining. The heart sections were incubated with primary α-actinin antibody and the corresponding second antibody, then imaged under an invert fluorescence microscope (Leica, Germany).

**Membrane potential measurements**

Mitochondrial membrane potential was detected through a Mitochondrial Membrane Potential JC-1 Kit (Beyotime Biotechnology, China) according to manufacturer's instructions. In brief, after the treatment, cells were washed with PBS for three times and then loaded with 5 μM JC-1 staining probe for 30 min at 37°C. Thereafter, the fluorescent images were analyzed under an invert fluorescence microscope.

**Analysis of the cell Oxygen Consumption Rate (OCR)**

The cell OCR was detected by a Seahorse XF-24 flux analyzer (Seahorse Biosciences, Agilent, USA) using a mitochondrial stress test (MST) assay kit (Seahorse Biosciences, Agilent, USA) according to the manufacturer's instructions. After different treatments, cells in each group were separately collected by trypsinization and planted in XF-24 extracellular flux assay plates with 2 × 10^4^ cells/well. After culturing at 37°C overnight, the cells were further changed to MST buffer after adherence at 37 °C for 2 h. Thereafter, 1 μM oligomycin, 1 μM FCCP, and 0.5 μM rotenone/antimycin A were subsequently added into each well for further experiments, respectively. Values of OCR (pmol O_2_/min) for each well were recorded and averaged for data analysis.

**Actinomycin D treatment**

Cellular transcription was inhibited with actinomycin D. After pretreatment with actinomycin D (0.1 μg/mL, MedChemExpress, NJ, USA), MCM cells were incubated with or without cEVs (1.0 × 10^9^ particles/mL).

**Immunofluorescence (IF) and immunohistochemical (IHC) Staining.**

For IF and IHC staining, cells or heart sections were incubated with rabbit anti-CD45, rabbit anti-CD31, rabbit anti-α actinin (Abcam, USA), rabbit anti-Vimentin (Abcam, USA), rabbit anti-4-HNE (Abcam, USA), rabbit anti-ATP5a1(Abclonal, China), rabbit anti-Tom20 (Abclonal, China) at 4°C overnight. Then, cells or sections were washed with PBS and incubated with the corresponding fluorescein-isothiocyanate-conjugated secondary antibodies (Cell Signaling Technology, USA) at room temperature for 1 hour. After staining the nuclei with DAPI (SouthernBiotech, USA), the samples were observed under a microscope (Leica, Wetzlar, Germany).

**Supplemental Results**

**
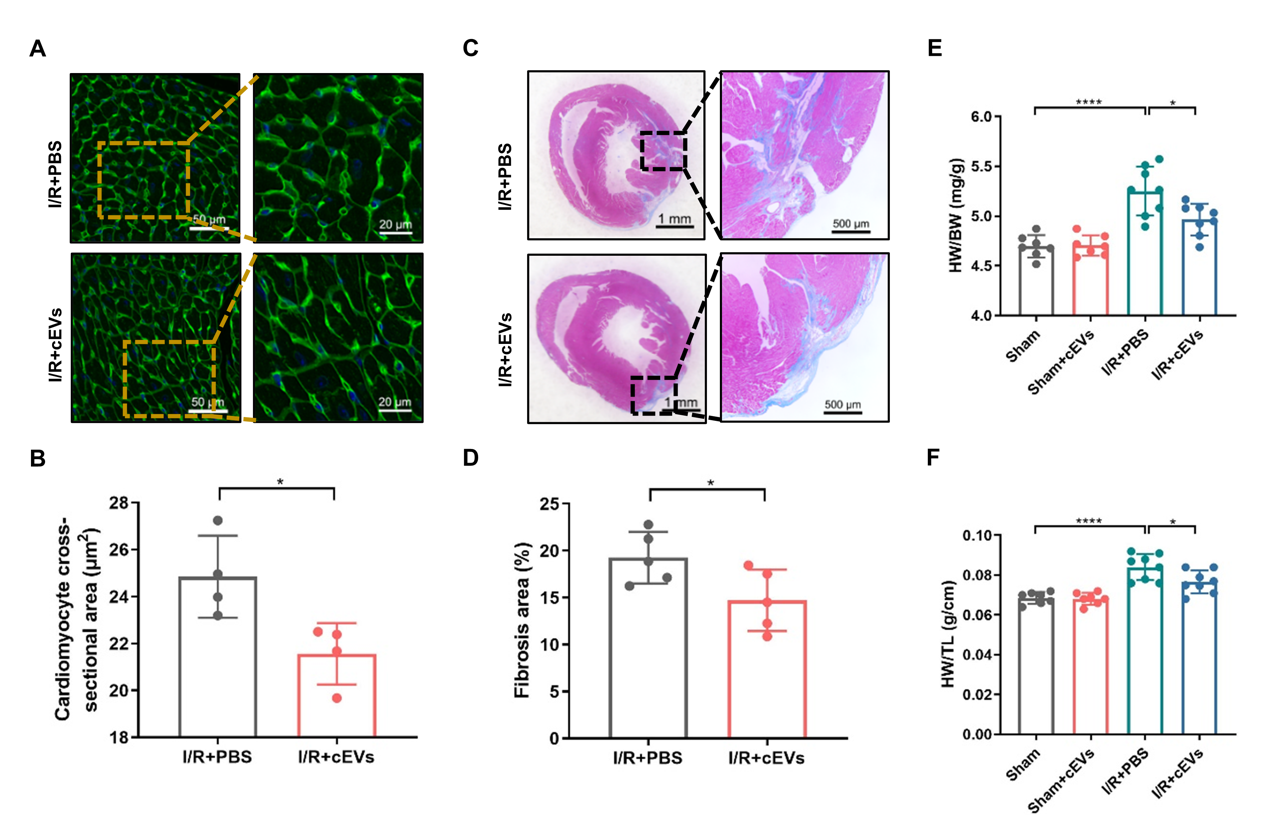
**

**Fig. S1** **cEVs alleviated cardiac remodeling in mice at 28 days after MI/R operation**

**A**, **B** WGA staining of cardiomyocytes cross-sectional area in mice and corresponding quantification analysis (n=4). **C, D** Masson trichrome staining showing cardiac fibrosis in mice at day 28 post MI/R and corresponding quantification analysis (n=5). **E, F** HW/BW (**E**) and HW/TL (**F**) in mice at day 28 post MI/R and corresponding quantification analysis (n=6-8). (Data were expressed as Mean±SD. Statistically significant: *p<0.05, ****p<0.0001).

**
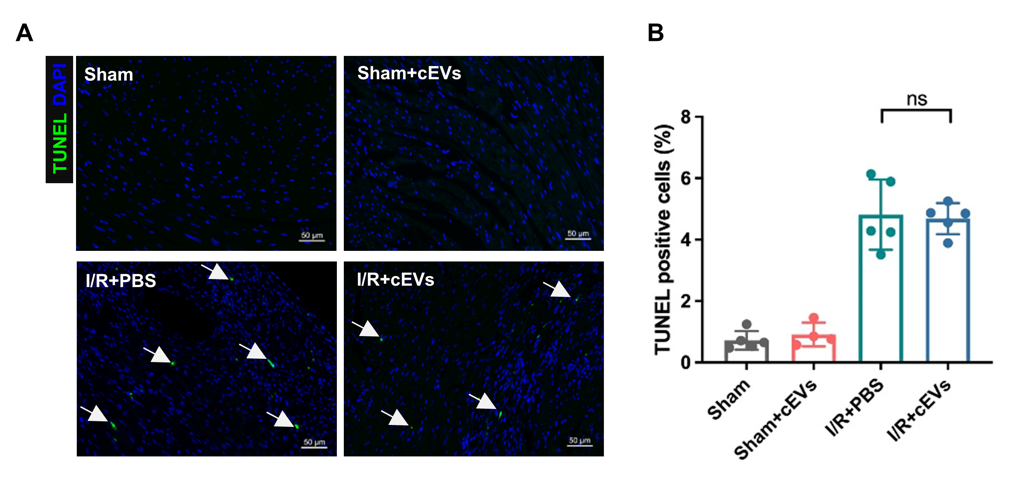
**

**Fig. S2** **TUNEL staining of heart sections in mice at 3 days after MI/R operation**

**A, B** Representative images (**A**) and quantification (**B**) of TUNEL staining of heart sections from mice with cEVs or PBS administration 3 days after MI/R operation. Scale bars: 50 μm (n =4-5). (Data were expressed as Mean±SD. Statistically significant: ns: not statistically significant)


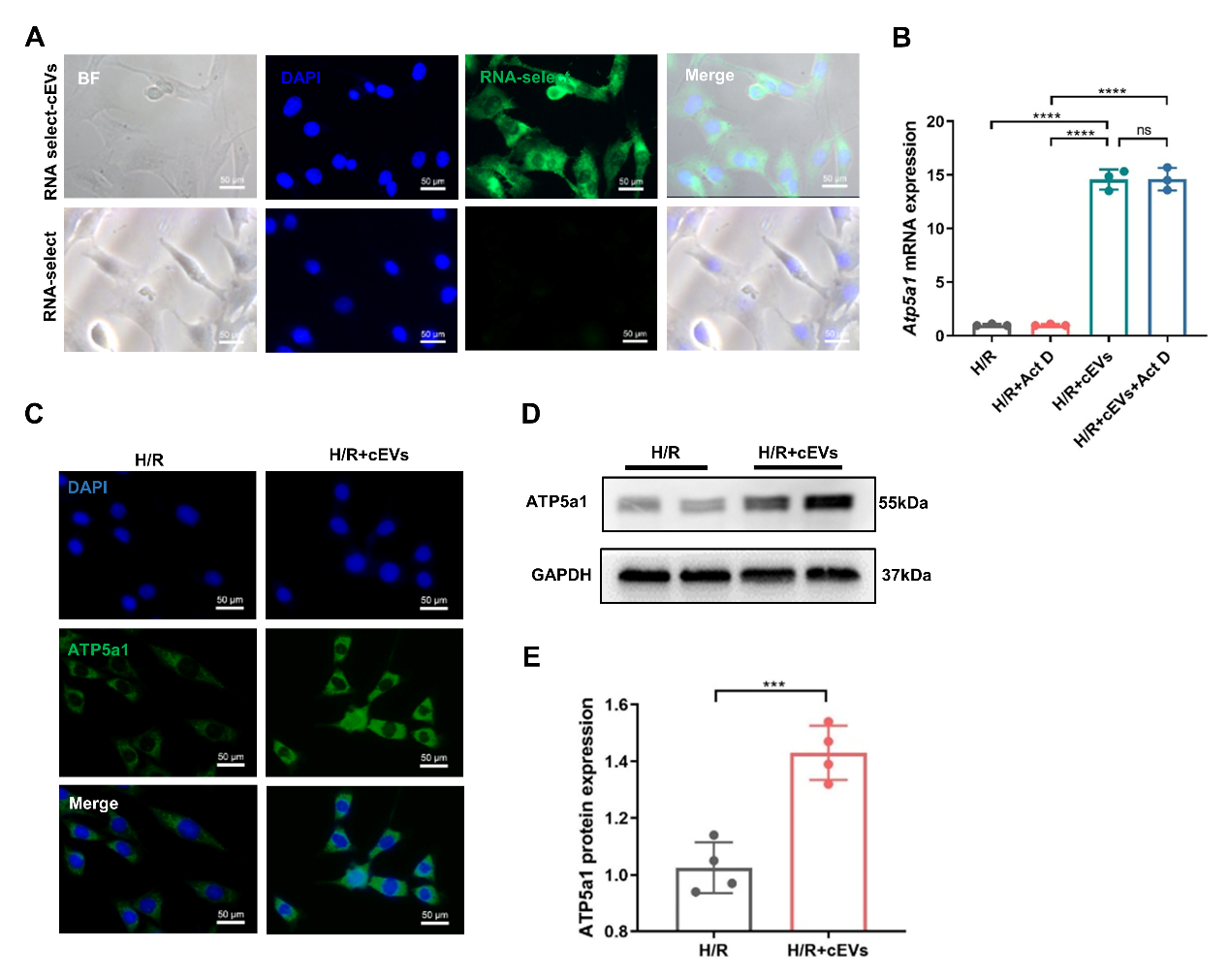


**Fig. S3 cEVs mediated transporting of ATP5a1 into cardiomyocytes**

**A** Representative micrograph showing cEVs transporting RNA into MCM cells. DiL was used to label cEVs, RNA-select was used to label RNA in cEVs, DAPI was used to label nuclei (scale bar = 50 μm).

**B** Representative ATP5a1 mRNA expression in cEVs treated MCM cells with or without Actinomycin D pretreatment (n=3). **C** Representative micrographs of ATP5a1 IF staining showing cEVs treatment increased ATP5a1 in MCM cells (scale bar = 50 μm). **D, E** Relative ATP5a1 protein expression in MCM cells after cEVs treatment detected using western blot and corresponding quantification analysis (n=3). (Data were expressed as Mean±SD. Statistically significant: ns: not statistically significant, ***p<0.001，****p<0.0001).


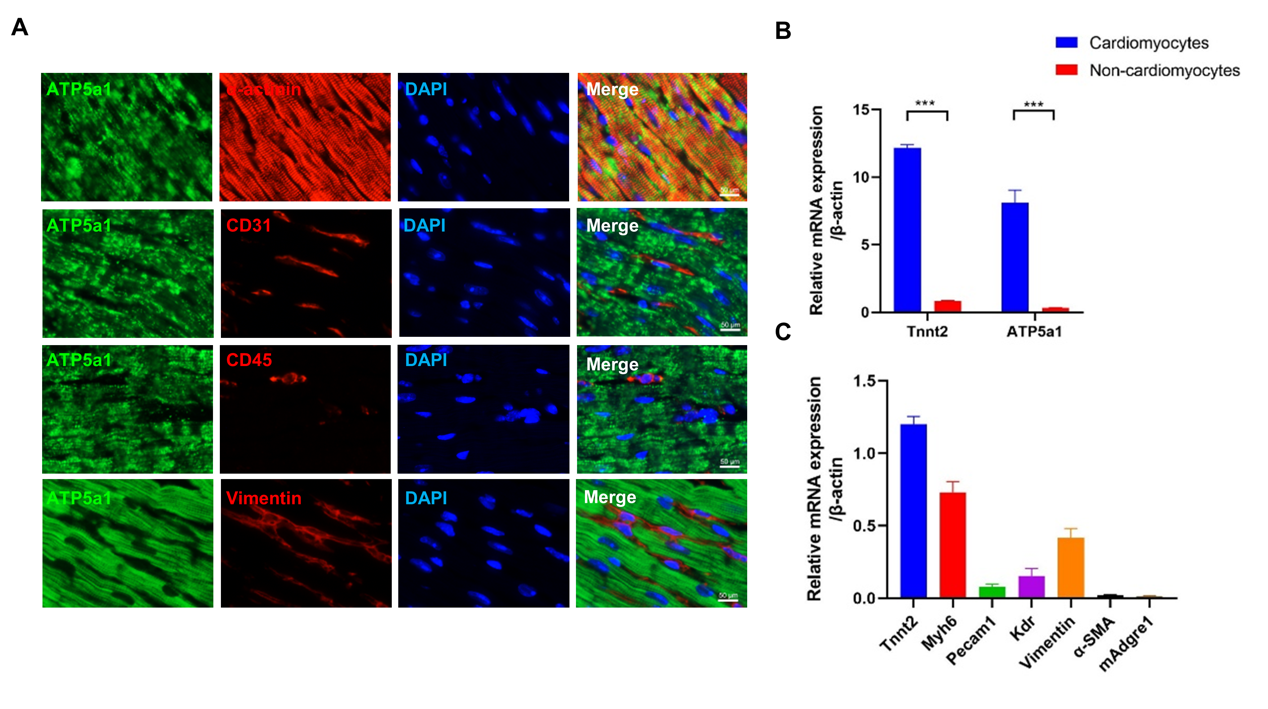


**Fig.** **S4 ATP5a1 in cEVs is mainly derived from cardiomyocytes**

**A** Representative micrograph of IF colocalization of ATP5a1 and different cellular markers in cardiac tissue (including α-actinin, CD31, CD45, and Vimentin) (scale bar = 50 μm). **B** Relative mRNA expression of Tnnt2 and ATP5a1 in cardiomyocytes and non-cardiomyocytes isolated from 6-8 weeks old mice heart (n=3). **C** Relative mRNA expression of Tnnt2, Myh6, Pecam1, Kdr, Vimentin, α-SMA, and mAdgre1 in cEVs detected by RT-qPCR (n=3). (Data were expressed as Mean±SD. Statistically significant: ***p<0.001).


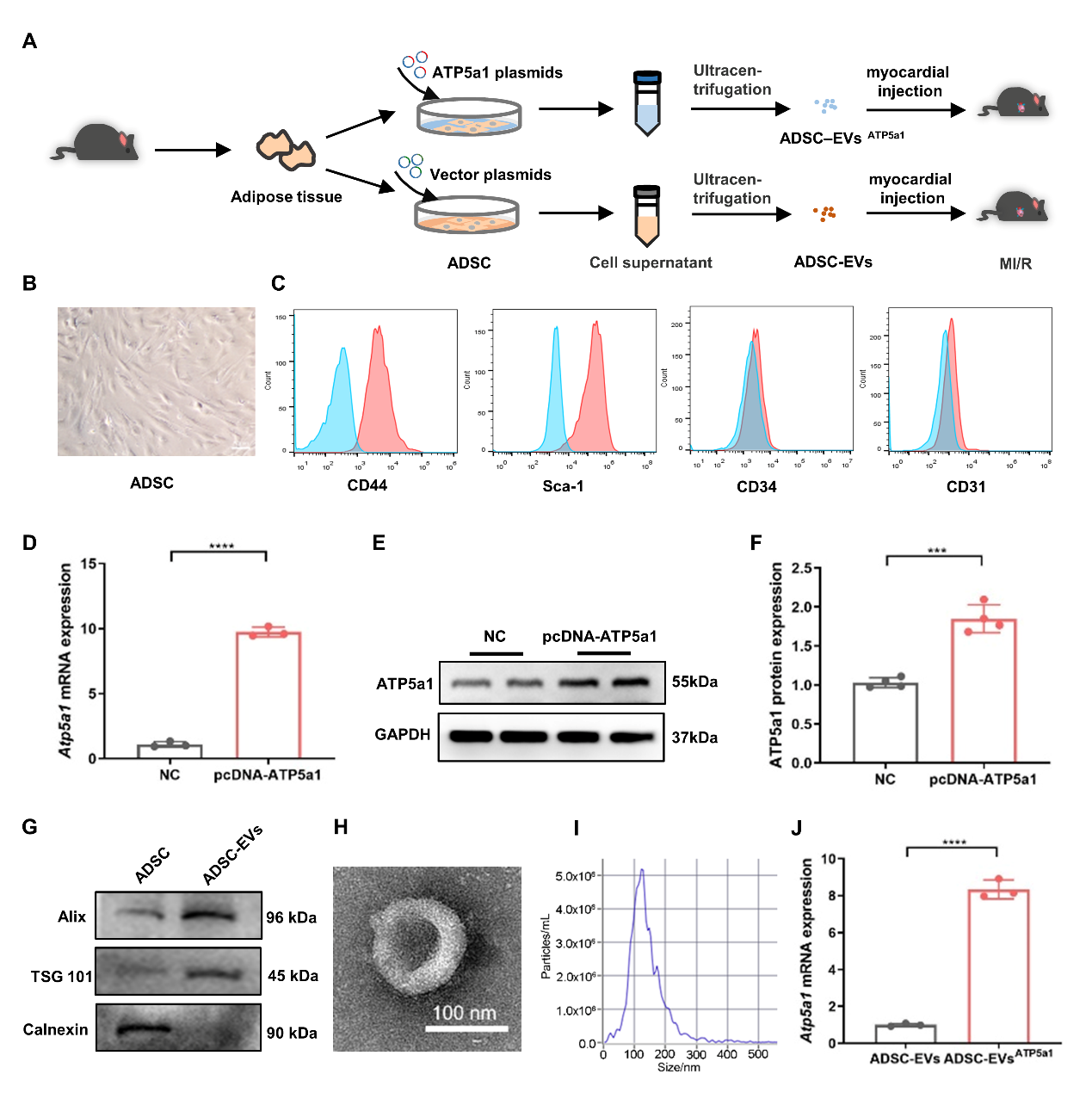


**Fig. S5 Overexpression of ATP5a1 in ADSC-derived EVs**

**A** Schematic diagram showing the culture of ADSC and isolation of ADSC derived EVs with overexpression of ATP5a1. **B** Morphology of ADSC observed under an inverted microscope (scale bar = 50 μm). **C** Phenotypic analysis of ADSC determined by flow cytometry showing ADSC was positive for CD44 and Scar-1, while negative for CD34 and CD31. **D** Relative mRNA expression of ATP5a1 in ADSC after transfection of ATP5a1 plasmid (n=6). **E, F** Relative protein expression of ATP5a1 in ADSC after transfection of ATP5a1 plasmid and corresponding quantification analysis (n=6). **G** Protein markers analysis of the ADSC derived EVs (TSG101, Alix, and Calnexin). **H** TEM images of ADSC derived EVs showing the expected cup shape (scale bar= 50 nm). **I** Size distribution for ADSC derived EVs detected by NTA. **J** Relative mRNA expression of ATP5a1 in ADSC derived EVs detected by RT-qPCR (n=3). (Data were expressed as Mean±SD. Statistically significant: ***p<0.001, ****p<0.0001).

**Table S1**: Primer sequences used for quantitative real-time PCR

| Gene | Forward primer (5′-3′) | Reverse primer (5′-3′) |
| --- | --- | --- |
| Actin | ATGACCCAAGCCGAGAAGG | CGGCCAAGTCTTAGAGTTGTTG |
| Ptgs2 | ACCTCTCTGAACTATGGTGTGAAC | CATGCTTGGGTCAGTCAATATTGG |
| TFAM | GCAACCCTCCTTTCTCCCAG | GATGGTGGTGGTGAGGCAAG |
| PGC-1α | GCCATTGCCTTCATGCTGTG | AGGTTCCCTCTCTGCTGCTT |
| ATP5a1 | GGAGCCCAGCAAGATCACAA | GGTGACAGTGACAGGGCTTT |
| Mt-co1 | TATGTTCTATCAATGGGAGC | GTAGTCTGAGTAGCGTCGTG |
| Mt-ND1 | CTAACAACTATTATCTTCCTAGGAC | GATGTATAAGTTGATCGTAACGG |
| Mt-Cytb | TGCTTTGAGGTATGAAGGAAAGG | ACATACTAGGAGACCCAGACAAC |
| Mt-ND6 | TGG TTG TCT TGG GTT AGC ATT | CGA TCC ACC AAA CCC TAA AA |
| Mt-ND4 | TCCTCCCTACTATGCCTAG | AGCATTCGGAGACAACAG |
| Mt-ND2 | AACCCACGATCAACTGAAGC | GGGCGAGGCCTAGTTTTATG |
| Mt-ND5 | TCTCTACATCAAGCCAACT | GATTGAGCCAGAGCATAT |
| Cox6b1 | GGAGTCAGGATGGCTGAAGA | ACCACTCACACACGGAGACA |
| Cox6c | GTCTGCGGGTTCATATTGCT | CAGCCTTCCTCATCTCTTCG |
| Atp5b | TCCACTGGACTCCACCTCTC | ATCTTTCTTGCCCGGGACAC |
| Cox8a | AGGGAGCAGTCTTCCCTCAT | ACCATGGAGACATCGAGAGG |
| Cox4i1 | CAGTTGTACCGCATCCAGTTT | GGATGGGGCCATACACATAG |
| Pkm | CCCCTCCCCTATCCTTTCCA | GTCCTGCATTCCTCCTCCAC |
| Ldha | GACCACACCCTTCTCGTCTG | CAGGCTCACAGGGGTAATCG |
| Tnnt2 | CAGAGCGGAAGAGTGGGAAG | GGTCGAACTTCTCAGCCTCC |
| Myh6 | CCTGGAGCGCATGAAGAAGA | CCCTTCACCGACTCTGCATT |
| Pecam1 | ACGCTGGTGCTCTATGCAAG | TCAGTTGCTGCCCATTCATCA |
| Kdr | TTTGGCAAATACAACCCTTCAGA | GCAGAAGATACTGTCACCACC |
| Vimentin | TTGCCCTTGAAGCTGCTAACT | GAAATCCTGCTCTCCTCGCC |
| α-SMA | CCTCCTTTGGCCAACATCCA | GACACCCTTGGCTTCCTCATC |
| mAdgre1 | AACATGCAACCTGCCACAAC | TTCACAGGATTCGTCCAGGC |

**References**

1. Ackers-Johnson M, Li PY, Holmes AP, O'Brien SM, Pavlovic D, Foo RS. A Simplified, Langendorff-Free Method for Concomitant Isolation of Viable Cardiac Myocytes and Nonmyocytes From the Adult Mouse Heart. Circ Res. 2016;119(8):909-20.

2. Nagata H, Ii M, Kohbayashi E, Hoshiga M, Hanafusa T, Asahi M. Cardiac Adipose-Derived Stem Cells Exhibit High Differentiation Potential to Cardiovascular Cells in C57BL/6 Mice. Stem Cells Transl Med. 2016;5(2):141-51.
